# Cholesterol metabolism reprogramming: shaping tumor immunity and immunotherapy

**DOI:** 10.1186/s13046-026-03791-7

**Published:** 2026-07-29

**Authors:** Jing-Wen Meng, Yue-Tao Tan, Shan-Mei Chen, Ting Li, Hui Wang

**Affiliations:** 1 Department of Radiation Oncology, The Affiliated Cancer Hospital of Xiangya School of Medicine, Central South University, Hunan Cancer Hospital, Changsha, 410013 P. R. China; 2 Institute of Biochemistry and Molecular Biology, Guangdong Provincial Key Laboratory of Medical Immunology and Molecular Diagnostics, School of Basic Medical Sciences, Guangdong Medical University, Dongguan, 523808 P. R. China; 3 Department of Pathology, The Affiliated Cancer Hospital of Xiangya School of Medicine, Central South University, Hunan Cancer Hospital, Changsha, 410013 P. R. China; 4 Department of Gastroenterology, School of Medicine, The Affiliated Cancer Hospital of Xiangya, Central South University, Hunan Cancer Hospital, Changsha, 410013 P. R. China; 5 Hunan Key Laboratory of Translational Radiation Oncology, The Affiliated Cancer Hospital of Xiangya School of Medicine, Central South University, Hunan Cancer Hospital, Changsha, 410013 P. R. China

**Keywords:** Cholesterol metabolism, Immune microenvironment, Immunoregulation, Tumor immunotherapy

## Abstract

Cholesterol, a vital biomolecule with essential physiological functions, undergoes frequent reprogramming of its metabolic pathways during tumorigenesis and progression. This reprogramming enables it to participate in immune regulation through multiple mechanisms. Accumulating evidence reveals the aberrant activation of cholesterol metabolic pathways across various malignancies. This dysregulation is characterized by enhanced synthesis, increased uptake, reduced efflux, and the accumulation of derivatives, such as cholesteryl esters and oxysterols. These alterations suggest that targeting cholesterol metabolism represents a promising strategy to enhance the efficacy of tumor immunotherapy. In cancer cells, reprogrammed cholesterol metabolism supports proliferation, stemness, and resistance to cell death, including ferroptosis and autophagy. Conversely, in immune cells, it modulates effector functions, polarization states, and the expression of immune checkpoints like PD-L1. Collectively, these effects shape an immunosuppressive tumor microenvironment that facilitates immune evasion. Furthermore, cholesterol metabolism intersects with critical oncogenic signaling pathways and is fine-tuned by non-coding RNAs, thereby further driving tumor progression and therapy resistance. This review systematically delineates the regulatory landscape of cholesterol homeostasis in both malignant and immune cells, highlighting its multifaceted impact on tumor immunity and therapeutic responses. A deeper understanding of cholesterol and its metabolite dynamics within the tumor microenvironment, as well as their crosstalk with other signaling networks, will not only aid in optimizing current immunotherapeutic approaches but also provide a crucial conceptual foundation for developing novel combination strategies.

## Introduction

Cholesterol, a derivative of the steroid nucleus, is classified as a sterol based on its specific chemical structure characterized by variations in carbon chain length and functional substituents. The human body contains approximately 140 g of cholesterol, which is widely distributed across all tissues primarily in the forms of free cholesterol and cholesteryl esters (CE) [1]. As an amphipathic lipid molecule, cholesterol serves as a fundamental structural component of cellular membranes. It contributes to membrane biogenesis and repair while maintaining optimal fluidity and stability [2, 3]. Moreover, cholesterol is integral to membrane-based signal transduction, a process that critically regulates cellular morphology and physiological functions [4, 5]. The metabolism of cholesterol constitutes a complex physiological process involving multiple biochemical pathways. The primary catabolic route for cholesterol occurs in the liver, where it is converted into bile acids [6]. Furthermore, cholesterol serves as an essential precursor for the biosynthesis of steroid hormones (e.g., corticosteroids and sex hormones) and vitamin D. Through specific enzymatic transformations, cholesterol is metabolized into these critical molecules, thereby playing a pivotal role in endocrine regulation and calcium homeostasis [7]. In summary, cholesterol underpins multiple essential physiological functions. Its synthesis and metabolism are maintained in a dynamic equilibrium, which is crucial for supporting diverse biological activities, including growth and development, substance transport, and cellular homeostasis.

Fueled by groundbreaking advances in tumor metabolism research, cholesterol metabolic reprogramming has emerged as a hallmark metabolic feature of cancer [8–10]. This review first elucidates the regulatory mechanisms of cholesterol homeostasis under physiological conditions and its reprogramming in tumors. It then thoroughly dissects the multifaceted impact of cholesterol metabolism on the tumor immune microenvironment and the functionality of various immune cells. Furthermore, the discussion extends to its mechanistic roles in emerging processes such as ferroptosis and autophagy. Finally, we provide a comprehensive overview of therapeutic strategies targeting cholesterol metabolism and evaluate their clinical translation prospects. By integrating these aspects, this review aims to establish a systematic theoretical framework that elucidates the role of cholesterol metabolism in tumor immunoregulation and therapy. This framework is intended to provide strategic insights for the development of innovative combination treatment strategies.

## Cholesterol biosynthesis and metabolism in normal organisms

### *De novo* cholesterol biosynthesis

Cholesterol in the body is derived from two primary sources: dietary intake and endogenous biosynthesis. In humans, most cholesterol (> 70%) is derived from *de novo* biosynthesis, this pool is classified as endogenous cholesterol. Dietary cholesterol constitutes a secondary source (approximately 25%), which is termed exogenous cholesterol. Cholesterol synthesis occurs in nearly all tissues, with the notable exceptions of adult neural tissue and mature erythrocytes. The liver (approximately 80%) and the small intestine (approximately 10%) are the predominant sites of synthesis. The biosynthesis of cholesterol utilizes acetyl-CoA as the fundamental substrate. The synthesis of a single cholesterol molecule requires 18 molecules of acetyl-CoA, 36 molecules of ATP, and 16 molecules of NADPH. Acetyl-CoA and ATP are primarily generated through the aerobic oxidation of glucose, whereas NADPH is supplied predominantly via the pentose phosphate pathway. Since cholesterol synthesis takes place in the cytosol and the smooth endoplasmic reticulum (ER), acetyl-CoA generated in the mitochondria must be transported to the cytosol via the citrate-pyruvate shuttle.

The cholesterol biosynthetic pathway comprises five distinct stages: (1) mevalonate synthesis, (2) isoprenoid synthesis, (3) farnesyl pyrophosphate (FPP) synthesis, (4) squalene formation, and (5) cyclization of squalene to cholesterol. These stages involve significant alterations in carbon chain length and molecular complexity, with enzymatic reactions occurring in compartmentalized cellular environments. This process exemplifies precise metabolic coordination between mitochondrial energy production and cytosolic biosynthesis. Stage 1: mevalonate synthesis. The initial stage involves the condensation of three acetyl-CoA molecules to form mevalonate. Initially, two acetyl-CoA molecules are condensed to acetoacetyl-CoA by thiolase. Acetoacetyl-CoA then condenses with a third acetyl-CoA molecule, catalyzed by 3-hydroxy-3-methylglutaryl-CoA (HMG-CoA) synthase, to form HMG-CoA. Subsequently, HMG-CoA is reduced to mevalonate by HMG-CoA reductase (HMGCR). This reduction catalyzed by HMGCR constitutes the rate-limiting step of the entire cholesterol biosynthesis pathway [11]. Statins, a class of lipid-lowering medications, exert their therapeutic effect by specifically inhibiting HMGCR activity, thereby suppressing cholesterol synthesis. Stage 2: isoprenoid synthesis. In this stage, mevalonate undergoes a series of phosphorylation and decarboxylation steps to form the five-carbon activated isoprene unit, isopentenyl pyrophosphate (IPP). IPP can then isomerize to dimethylallyl pyrophosphate. Both IPP and dimethylallyl pyrophosphate serve as activated building blocks for subsequent condensation reactions. The pyrophosphate moiety acts as a high-energy group, endowing these intermediates with the chemical potential required to drive the ensuing macromolecular assembly. Stage 3: FPP synthesis. In this stage, one molecule of IPP and one molecule of dimethylallyl pyrophosphate undergo head-to-tail condensation to form the C10 intermediate, geranyl pyrophosphate. Geranyl pyrophosphate then undergoes a second head-to-tail condensation with another IPP molecule to yield the C15 product, FPP. Notably, FPP serves as a crucial metabolic branch point, acting not only as a direct precursor for cholesterol but also as an essential intermediate for synthesizing dolichol and ubiquinone (coenzyme Q) [12]. Stage 4: squalene formation. This stage involves the reductive, head-to-head condensation of two FPP molecules, catalyzed by squalene synthase, to form squalene. This reaction produces the linear C30 triterpene hydrocarbon, squalene, which is characterized by its unsaturation with six double bonds [13]. Stage 5: cyclization to cholesterol. This final stage encompasses the conversion of squalene to cholesterol. Squalene is first cyclized to form lanosterol, which then undergoes approximately 20 additional enzymatic reactions to yield cholesterol. The committed step in this stage is the epoxidation of squalene to squalene-2,3-epoxide, catalyzed by squalene monooxygenase (SQLE) [14]. This epoxide serves as the immediate precursor for cholesterol synthesis in animals. In plants and fungi, it is channeled through distinct pathways to yield sterols such as stigmasterol and ergosterol, respectively. Given its specificity to the cholesterol biosynthetic pathway, SQLE has emerged as a novel and promising pharmacological target for therapeutic intervention.

### Cholesterol uptake pathways

In addition to *de novo* biosynthesis, cells acquire exogenous cholesterol through dietary sources and via systemic circulation. Dietary cholesterol is primarily derived from animal-based foods, including eggs, organ meats, and seafood. In humans, cholesterol absorption occurs predominantly in the small intestine [15], where intestinal epithelial cells mediate the process via the transmembrane protein Niemann-Pick C1 like 1 (NPC1L1). NPC1L1 facilitates cholesterol uptake via clathrin-mediated endocytosis, thereby transporting dietary cholesterol from the intestinal lumen into enterocytes [16]. Studies in mice harboring mutations in the endocytic motifs of NPC1L1 have confirmed its critical role. Following cholesterol administration, mutant mice exhibited cholesterol accumulation at the brush border membrane compared to wild-type controls. Concurrently, these mutants displayed reduced cholesterol levels in both the liver and plasma, collectively confirming the indispensable role of NPC1L1 in intestinal cholesterol absorption [17]. Upon cholesterol binding, the NPC1L1-cholesterol complex undergoes clathrin- and adaptor protein 2-mediated endocytosis, which facilitates its vesicular transport into the cell. The cholesterol is subsequently delivered to the ER for esterification into CEs, while NPC1L1 recycles back to the plasma membrane. NPC1L1 is the molecular target of the lipid-lowering drug ezetimibe. Ezetimibe exerts its therapeutic effect by inhibiting the internalization of NPC1L1, thereby blocking intestinal cholesterol absorption and ultimately lowering systemic cholesterol levels [15]. Circulating cholesterol is primarily acquired by cells through receptor-mediated endocytosis of low-density lipoprotein (LDL). The liver, the principal site for cholesterol biosynthesis, packages endogenously synthesized and dietary cholesterol with apolipoproteins to form very-low-density lipoproteins (VLDLs), which are subsequently secreted into the bloodstream. In the circulation, VLDL particles undergo lipolysis and compositional changes, ultimately transforming into cholesterol-enriched LDLs [18]. The low-density lipoprotein receptor (LDLR) on the cell membrane specifically recognizes and binds to LDL particles. Following the formation of the LDLR-LDL complex, it is internalized into the cell via clathrin-mediated endocytosis. Within endosomes, the LDLR dissociates from the LDL particle and recycles back to the plasma membrane. The LDL particle is then transported to lysosomes for degradation. Within lysosomes, the CEs carried by LDL are hydrolyzed by acid lipase, releasing free cholesterol. The released free cholesterol is then transported across the lysosomal membrane into the cytoplasm by transporters such as lysosome-associated membrane glycoprotein 2 for cellular utilization [16]. Additionally, high-density lipoprotein (HDL) mediates reverse cholesterol transport from peripheral tissues to the liver. The class B scavenger receptor type I facilitates the selective uptake of CEs from HDL particles into hepatocytes [19].

### Cholesterol efflux and esterification

Cellular cholesterol efflux is a critical physiological process primarily mediated by ATP-binding cassette (ABC) transporters, including ABCA1, ABCG1, ABCG5, and ABCG8. This process is central to reverse cholesterol transport (RCT), a pathway through which excess cholesterol is removed from peripheral tissues and delivered to the liver for metabolism and excretion. The transcription factors liver X receptors (LXRs) function as master regulators of cholesterol efflux. Upon activation, LXRs bind to specific response elements in the promoter regions of target genes such as ABCA1 and ABCG1, upregulating their expression and thereby potentiating cellular cholesterol efflux capacity. ABCG5 and ABCG8 are predominantly expressed in hepatocytes and intestinal epithelial cells. They form functional heterodimers (ABCG5/ABCG8) that mediate the direct excretion of excess cholesterol into the bile canaliculi and the intestinal lumen [20]. Collectively, these regulatory mechanisms maintain intracellular cholesterol homeostasis. This homeostasis prevents pathological cholesterol accumulation, thereby mitigating the risk of atherosclerosis and related cardiovascular diseases.

In parallel to cholesterol efflux, esterification constitutes another critical mechanism for preventing the intracellular accumulation of free cholesterol. CEs serve as both the storage and transport forms of cholesterol. Their formation is primarily catalyzed by two key enzymes: acyl-CoA: cholesterol acyltransferase (ACAT) and lecithin-cholesterol acyltransferase (LCAT). ACAT, which catalyzes intracellular cholesterol esterification, exists in two mammalian isoforms: ACAT1 and ACAT2. Cholesterol acts as an allosteric activator of ACAT1 by directly binding to and stimulating its enzymatic activity. Activated ACAT1 subsequently promotes the esterification of free cholesterol in a positive feedback manner, facilitating its storage as cytoplasmic lipid droplets [21]. ACAT2 is predominantly expressed in hepatocytes and enterocytes, where it facilitates intestinal cholesterol absorption by catalyzing esterification. In contrast, LCAT is synthesized in the liver and secreted into the bloodstream, where it performs its functions. LCAT catalyzes the esterification of free cholesterol on HDL particles by transferring a fatty acid from the *sn-2* position of phosphatidylcholine (lecithin), yielding CE and lysolecithin. This reaction is crucial for HDL maturation, as it esterifies free cholesterol within nascent HDL particles, increasing their core lipid content and promoting structural remodeling. Furthermore, cholesterol esterification plays a vital role in systemic lipoprotein metabolism and transport, as CEs constitute essential structural components of chylomicrons, VLDLs, and LDLs. Thus, whereas cholesterol efflux removes excess cholesterol from cells, esterification facilitates its storage and transport. Together, these two mechanisms operate in a coordinated manner to maintain intracellular cholesterol homeostasis.

### Cholesterol oxidation and oxysterols

**Fig. 1:**
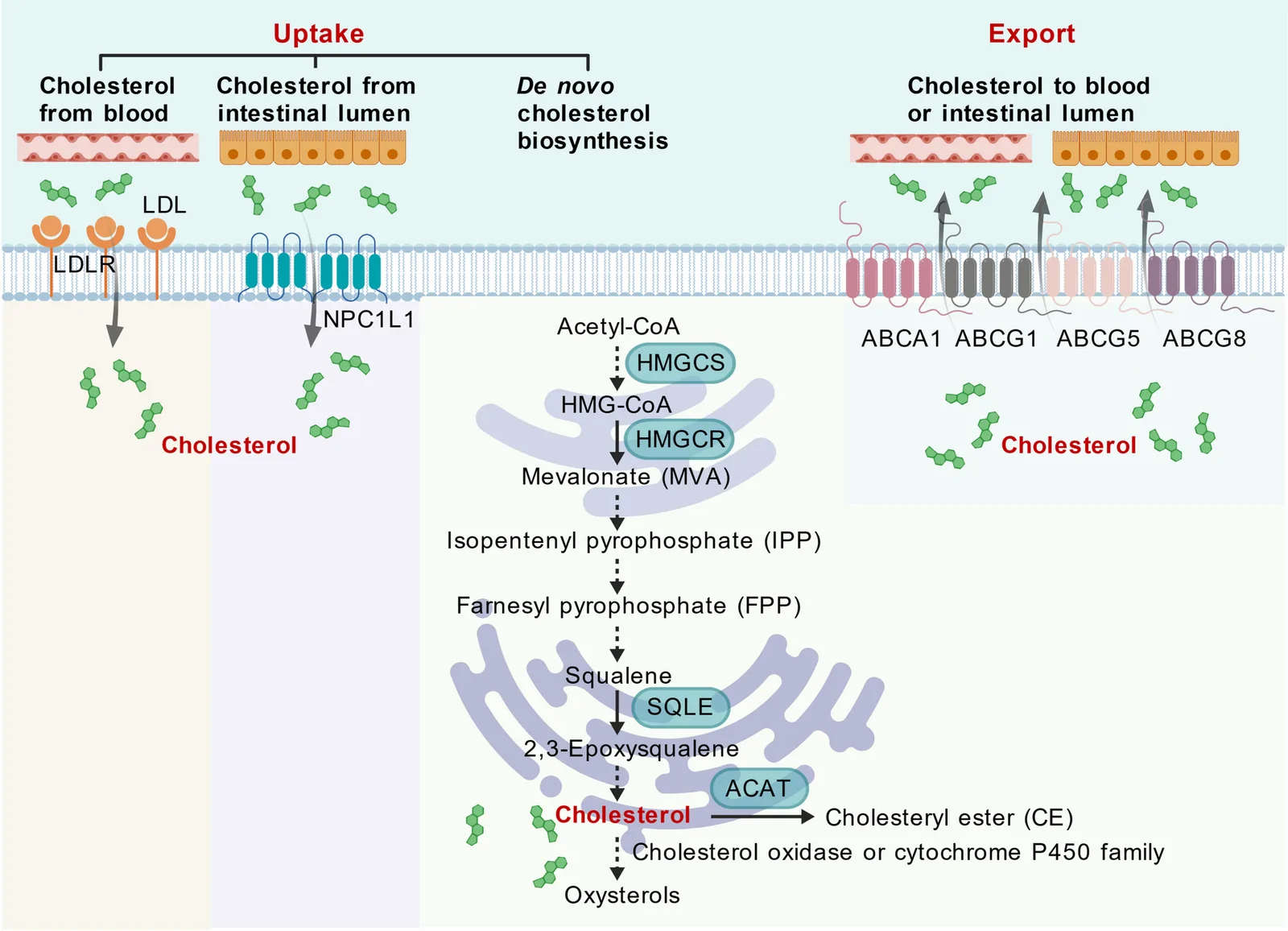
Cholesterol *de novo* synthesis, uptake, and efflux in normal cells. This diagram summarizes the entire metabolic network responsible for maintaining cholesterol homeostasis in normal cells. Cholesterol biosynthesis initiates with acetyl-CoA as the foundational substrate. Acetyl-CoA is first converted to HMG-CoA by HMG-CoA synthase. HMG-CoA is then reduced to mevalonate by HMGCR, which catalyzes the rate-limiting step of the entire pathway and serves as its major regulatory node. Through subsequent enzymatic steps, mevalonate is converted to IPP and FPP. SQLE then converts FPP into 2,3-epoxysqualene, leading to cholesterol formation after a series of additional reactions. Newly synthesized cholesterol can subsequently be esterified by ACAT into CE for storage within lipid droplets, or it can be oxidized by enzymes including cholesterol oxidases and cytochrome P450 monooxygenases to generate various oxysterols. Exogenous cholesterol is acquired via two distinct pathways: intestinal absorption relies on the NPC1L1 transporter, whereas circulating cholesterol gains cellular entry through LDLR-mediated endocytosis. To prevent accumulation, excess intracellular cholesterol is effluxed via specific membrane transporters, including ABCA1, ABCG1, ABCG5, and ABCG8, into the circulation or into the bile/intestinal lumen, contributing to cellular cholesterol homeostasis

Cholesterol oxidation is a crucial metabolic process encompassing both enzymatic and non-enzymatic reactions, which yield a diverse array of oxidized derivatives known as oxysterols. Enzymatic oxidation is primarily mediated by cholesterol oxidases (e.g., AOX1) and specific cytochrome P450 enzymes, such as CYP46A1, CYP27A1, and CYP7A1. They catalyze the site-specific hydroxylation of cholesterol (e.g., at the C-7 or C-27 position), yielding hydroxycholesterol derivatives. These primary oxysterols can subsequently undergo further transformations via redox reactions or other modifications, giving rise to a broader spectrum of metabolic derivatives [22]. In contrast, non-enzymatic oxidation, driven by reactive oxygen species (ROS) or free radicals, predominantly generates cytotoxic oxysterols, including 7-ketocholesterol (7-KC), 7β-hydroxycholesterol, and 5,6-epoxycholesterol [23]. As oxidized derivatives of cholesterol, oxysterols play significant and diverse roles in a wide range of physiological and pathological processes. Notably, specific hydroxycholesterols have been identified as endogenous ligands for LXRs. By directly binding to and activating LXRs, these oxysterols regulate the expression of cholesterol efflux-related genes, such as ABCA1 and ABCG1, thereby promoting RCT. 25-Hydroxycholesterol (25-HC), a biologically active oxysterol produced in response to cellular cholesterol overload and immune stimuli, functions as a key mediator in both innate and adaptive immune responses. It modulates immune cell functions and inflammatory signaling pathways through both LXR-dependent and LXR-independent mechanisms [24]. (Fig. 1).

## Cholesterol metabolic reprogramming in tumorigenesis and progression

Under normal physiological conditions, cholesterol metabolism is tightly regulated to maintain homeostasis. In cancer cells, however, this homeostasis is disrupted to meet the heightened metabolic demands of rapid proliferation, leading to significant reprogramming of cholesterol metabolism. A hallmark of this reprogramming is the frequent accumulation of cholesterol within tumors, which is driven by the altered expression of a suite of genes involved in cholesterol metabolism. Extensive research supports a dual role for cholesterol metabolic reprogramming in cancer: it is both a consequence of oncogenic transformation and an active driver of multiple cancer hallmarks [25]. Consequently, dysregulated cholesterol metabolism and tumor progression engage in a mutually reinforcing, feed-forward relationship. This reprogramming manifests in cancer cells as a coordinated shift towards enhanced *de novo* synthesis, increased exogenous uptake, and the accumulation of cholesterol and its derivatives. These alterations collectively fuel critical oncogenic processes, including sustained proliferation, evasion of cell death, metastatic dissemination, and adaptation to the tumor microenvironment (TME).

### Characteristics of cholesterol metabolism in tumors

Cellular cholesterol homeostasis is primarily governed by two key transcription factor families: the sterol regulatory element-binding proteins (SREBPs) and the LXRs. SREBPs promote cholesterol anabolism by activating genes involved in biosynthesis and uptake, thereby elevating intracellular cholesterol levels. Conversely, LXRs promote cholesterol catabolism and efflux by upregulating efflux transporters (e.g., ABCA1, ABCG1) and concurrently suppressing uptake pathways, thereby lowering intracellular cholesterol concentrations [26]. Moreover, LXR can also induce the transcription of other target genes, including SREBPs, to regulate cholesterol metabolism [27]. Tumor cells frequently upregulate cholesterol biosynthetic pathways to enhance cholesterol production. This upregulation supplies the essential cholesterol required for robust membrane biogenesis and signaling, while also establishing a metabolic buffer that promotes survival under stress conditions, collectively fueling tumor cell proliferation [28, 29]. The SREBP family consists of two main isoforms: SREBP1 (with splice variants 1a and 1b), which primarily governs fatty acid synthesis, and SREBP2, the master regulator of cholesterol biosynthetic genes. SREBP2 exerts its function by binding to sterol regulatory elements in the promoters of key genes like HMGCR and SQLE, thereby enhancing their transcription. Under physiological conditions, a classic negative feedback loop maintains homeostasis: elevated intracellular cholesterol inhibits the proteolytic activation and nuclear translocation of SREBP2, thereby downregulating cholesterol synthesis and uptake [30]. In cancer, this regulatory circuit is frequently hijacked. Aberrant activation of oncogenic signaling pathways disrupts the normal feedback inhibition of SREBP2, leading to its constitutive activation and a consequent sustained upregulation of cholesterol synthesis and uptake. This dysregulation is commonly driven by mutations that hyperactivate the PI3K/AKT/mTORC1 axis across various cancer types, including activating mutations in growth factor receptors, such as epidermal growth factor receptor (EGFR) and human epidermal growth factor receptor 2 (HER2), oncogenic mutations in PIK3CA, or loss of the tumor suppressor phosphatase and tensin homolog (PTEN). This sustained PI3K/AKT/mTORC1 signaling promotes the proteolytic processing of SREBP2. It facilitates the Golgi transport of the SREBP cleavage-activating protein (SCAP)-SREBP2 complex and enhances the activities of Site-1 protease and Site-2 protease, ultimately increasing the production of transcriptionally active, mature SREBP2 [31, 32]. Following activation, mature SREBP2 translocates to the nucleus and binds to promoter regions of genes involved in cholesterol synthesis and uptake (including HMGCR and LDLR), enhancing their transcription and expression to upregulate cholesterol biosynthesis and acquisition. Notably, the tumor suppressor p53 acts as a brake on this pathway. It transcriptionally upregulates ATP-binding cassette transporter A1 (ABCA1), which promotes cholesterol efflux and indirectly inhibits SREBP2 activity, leading to suppression of mevalonate pathway genes. Consequently, loss-of-function mutations in p53 relieve this inhibition, thereby enhancing lipogenesis and cholesterol synthesis in tumors [33].

Furthermore, tumors markedly accumulate cholesterol derivatives, notably CEs and oxysterols. This accumulation is driven by the upregulation of cholesterol esterification, heightened oxidative stress, and dysregulation of key metabolic enzymes. These accumulated metabolites subsequently fuel critical oncogenic processes, such as sustained proliferation, metastasis, and immune evasion. This phenomenon underscores a key metabolic adaptation employed by cancer cells to thrive within hostile TME. At the transcriptional level, ACAT1 expression is predominantly regulated by SREBP2 and hypoxia-inducible factor-1α (HIF-1α). The activity of these transcription factors directly links to enhanced CE accumulation. Activating mutations in oncogenes (e.g., MYC) and the inactivation of tumor suppressors (e.g., p53, PTEN) converge to enhance SREBP2 activity. This occurs via the PI3K/AKT/mTOR axis, which collectively upregulates ACAT1 and synergistically promotes CE accumulation. Under conditions of energy stress (e.g., hypoxia), HIF-1α indirectly upregulates ACAT1 transcription. This promotes CE storage, serving as a crucial adaptive response that helps tumor cells cope with metabolic challenges. This metabolic flexibility provides tumor cells with on-demand access to essential membrane components and potential energy sources, thereby supporting their rapid and sustained proliferation.

The accumulation of specific oxysterols, such as 27-hydroxycholesterol (27-HC) and 7-KC, within the TME stems from two primary sources: elevated ROS and the hyperactivation of cytochrome P450 enzymes, including CYP27A1 and CYP46A1. For instance, mitochondrial elongation factor 2 (MIEF2), a regulator of mitochondrial dynamics, enhances mitochondrial ROS production in ovarian cancer cells. This elevated ROS activates the AKT/mTOR signaling cascade, which upregulates SREBP1 and SREBP2, leading to the transcriptional activation of their target genes and a consequent boost in oxysterol biosynthesis and related lipid metabolic pathways. Clinically, high MIEF2 expression correlates with poor prognosis in ovarian cancer patients [34]. In the TME, the accumulation of oxysterols is closely associated with their structural and functional properties. Substantial experimental evidence demonstrates that accumulated oxysterols play critical roles in various malignancies. For example, 27-HC is highly expressed in estrogen receptor-positive breast tumors, where it promotes proliferation and growth by modulating estrogen receptor signaling (e.g., via genes like ARMC1 and PARD6B) and engaging the GDNF-RET pathway [35]. 25-HC induces anoikis in colon cancer DLD1 cells, yet in hepatocellular carcinoma (HCC), it promotes migration and intrahepatic metastasis by upregulating TLR4-dependent fatty acid-binding protein 4 [36]. Conversely, 7-KC triggers ER stress and promotes cell death in human colon cancer HT-29 cells by upregulating ER chaperones and membrane kinases [37]. Beyond direct effects on tumor cells, oxysterols also function as endogenous LXR ligands. LXR activation suppresses SREBP signaling, thereby inhibiting LDL uptake and promoting cholesterol efflux [24, 38]. For instance, the CYP27A1/27-HC axis can inhibit cell proliferation in an LXR-dependent manner [39]. In summary, oxysterols exhibit a complex and context-dependent duality in oncology, functioning as either pro-tumorigenic or anti-tumorigenic agents based on the specific cellular and molecular composition of the TME.

To satisfy the heightened cholesterol demand associated with tumorigenesis and progression, tumor cells exhibit increased HMGCR and LDLR activity alongside reduced ABCA1 levels. Multiple regulatory pathways, including p53-mediated signaling (associated with SREBP1 and ABCA1), LXR-mediated PI3K/AKT/SREBP1/LDLR signaling (involving LXR, SREBP1, and LDLR), and MAPK signaling (related to ACAT1 and SREBP1). This leads to enhanced cholesterol synthesis and uptake, reduced efflux, and accumulation of cholesterol derivatives such as CE and oxysterols, thereby promoting tumor progression or facilitating other cellular functions.

Furthermore, plasticity and diversity are hallmark features of the TME, where cholesterol metabolism is tightly integrated with other metabolic networks, notably glycolysis and fatty acid oxidation (FAO), to collectively shape cancer metabolic plasticity [40]. Citrate derived from glycolysis is exported from mitochondria to the cytosol via solute carrier family 25 member 1 (SLC25A1) and is subsequently converted to acetyl-CoA by ATP citrate lyase, which is then utilized for cholesterol synthesis [41]. In cholesterol-enriched tumor cells, this mitochondrial citrate efflux is markedly accelerated, meeting the demand for lipid precursors and thereby reinforcing the Warburg effect. Moreover, cholesterol upregulates key glycolytic enzymes including GLUT1, HK2, and PFK1 to promote glucose uptake and aerobic glycolysis. In turn, hyperactivated glycolysis stabilizes HIF-1α, which transcriptionally activates SREBP2 and LDLR, further enhancing cholesterol uptake and synthesis and thereby establishing a positive feedback loop. Studies have demonstrated that, in endometrial cancer cells, hyperglycemia promotes the transcription of the rate-limiting enzymes of the glycolysis-cholesterol synthesis axis, HK2 and HMGCS1, in an estrogen-related receptor α-dependent manner, thereby suppressing the autophagy-lysosomal pathway, modulating the pyroptosis signaling pathway, and ultimately conferring drug resistance [42]. Concurrently, cellular cholesterol accumulation upregulates the lipid scavenger receptor CD36 to augment exogenous fatty acid uptake, whereas cholesterol-derived oxysterols transcriptionally upregulate the rate-limiting enzyme for mitochondrial fatty acid transport, CPT1A, via PPARα activation to enhance FAO flux. Collectively, cholesterol metabolism does not operate as an isolated pathway but rather as an integrated component of a dynamic metabolic network. This interconnectedness carries profound therapeutic implications, as isolated targeting of cholesterol synthesis may be counterbalanced by compensatory upregulation of FAO or glycolysis, which is a metabolic adaptation under therapeutic stress. Therefore, combination strategies that simultaneously target multiple metabolic axes are required to coordinate adaptive responses across divergent TME and treatment pressures, thereby providing a theoretical foundation for rational personalized metabolic intervention [40].

### The intrinsic drivers of cholesterol metabolism

In cancer cells, the aberrant activation of oncogenes coupled with the inactivation or mutation of tumor suppressor genes constitute fundamental drivers of cholesterol metabolic reprogramming. Dysregulated oncogenes thus serve a dual function: they drive malignant transformation and proliferation while simultaneously reprogramming cholesterol metabolism to meet the heightened demands of tumors for energy, membrane biogenesis, and signaling molecules. For instance, in glioblastoma (GBM), the oncogene MYC upregulates genes in the mevalonate pathway. The resulting metabolite, mevalonate, in turn activates miR-33b, creating a positive feedback loop that further amplifies MYC expression [43]. Across diverse tumor types, aberrant activation of key signaling pathways, such as the RTK/RAS/RAF/MEK cascade and the PI3K/AKT/mTOR axis, converges to activate SREBP transcription factors, thereby promoting intracellular cholesterol synthesis and accumulation. In hepatocytes, transgenic expression of the oncogene c-Fos suppresses LXRα signaling, consequently enhancing the production of cholesterol and its derivatives [44]. Conversely, inactivating mutations in tumor suppressor genes disrupt canonical functions like cell cycle control and genome maintenance, and additionally propel tumor progression by remodeling cholesterol metabolism. For example, loss-of-function mutations or deletions in TP53 can abrogate its suppressive effect on the mevalonate pathway, leading to upregulated cholesterol synthesis [33]. Loss of the tumor suppressor PTEN induces hyperactivation of the PI3K/AKT pathway. This, in turn, upregulates the LDLR via SREBP activation, culminating in the intracellular accumulation of CEs. In summary, aberrant activation of oncogenic signaling pathways acts as a primary driver of cholesterol metabolic reprogramming. Concurrently, the loss or mutation of key tumor suppressor genes relieves the inherent negative regulation on this metabolism. These events often operate synergistically, establishing a permissive and self-reinforcing metabolic state that fuels tumor progression.

### Regulation of cholesterol metabolism by the tumor microenvironment

The TME actively shapes cholesterol metabolic reprogramming through a confluence of factors, notably hypoxia, acidosis, and inflammation. The rapid and uncontrolled proliferation of tumor cells creates an oxygen-depleted milieu, establishing hypoxia as a nearly universal hallmark of the TME. Under hypoxic conditions, HIF-1α directly or indirectly activates the SREBP2 pathway, leading to the transcriptional upregulation of genes governing cholesterol synthesis and uptake [45]. Furthermore, acidosis, which is driven by the accumulation of lactic acid and H⁺ ions from hypoxia and enhanced glycolysis, constitutes another defining feature of the TME. At pH 6.5, CD36 expression on the plasma membrane of cancer cells increases [46], thereby enhancing their capacity for exogenous cholesterol uptake. When the extracellular pH drops to 6.8, it facilitates the nuclear translocation of SREBP2, activating its downstream transcriptional program [47]. Acidosis can also trigger the activation of the PI3K/AKT/mTOR signaling pathway, contributing to cholesterol metabolic reprogramming in liver cancer cells [48]. Within the inflammatory TME, the bacterial product lipopolysaccharide activates the NF-κB signaling pathway by engaging components such as IκB kinase α and TAK1-binding protein 3. This activation subsequently stimulates cholesterol biosynthesis and uptake. Moreover, elevated cholesterol levels can further reinforce NF-κB signaling through a positive feedback loop [49]. Additionally, the rapid proliferation of cancer cells requires substantial nutrient acquisition from the TME, resulting in a nutrient-depleted state within the TME. Both hypoxia and nutrient scarcity within the TME are potent inducers of ER stress [50, 51]. Given that the ER is the primary site for cholesterol biosynthesis, esterification, and oxidation, ER stress directly perturbs these key metabolic processes.

### Non-coding RNAs and cholesterol homeostasis in cancer

In recent years, non-coding RNAs (ncRNAs), including microRNAs (miRNAs), long non-coding RNAs (lncRNAs), and circular RNAs, have emerged as key epigenetic regulators of cholesterol metabolism in cancer, intricately controlling its synthesis, uptake, efflux, and oxidative modification [52–55]. By modulating this cholesterol metabolic reprogramming, ncRNAs play a pivotal role in driving cancer progression. Among these, miRNAs are prominent regulators of cholesterol homeostasis and transport. For instance, miR-33a, which resides within an intron of the SREBF2 gene, functions as an oncogenic miRNA. It post-transcriptionally represses ABCA1 expression by binding to its 3’ untranslated region, thereby impairing HDL biosynthesis and cholesterol efflux in various cancers. Other miRNAs, including miR-223 and miR-320b, similarly target ABCA1 to regulate cholesterol efflux [56–58]. The lncRNA lncARSR promotes tumor progression in HCC and renal cell carcinoma by regulating AKT signaling and modulating SREBP2 expression [59]. Similarly, the lncRNA lnc030 is highly expressed in breast cancer stem cells, where it functions as a critical regulator of breast tumorigenesis and progression. It interacts with poly(rC)-binding protein 2 to stabilize SQLE mRNA, thereby increasing cholesterol synthesis [60]. A hypoxia-responsive circular RNA, circINSIG1, is upregulated in colorectal cancer (CRC). It encodes a peptide, circINSIG1-121, which promotes the ubiquitination and degradation of INSIG1, which is a key negative regulator of cholesterol metabolism, by recruiting the CUL5-ASB6 E3 ubiquitin ligase complex. This leads to the induction of cholesterol biosynthesis, thereby facilitating CRC proliferation and metastasis [61]. In addition, lncRNAs and circRNAs can function as competing endogenous RNAs (ceRNAs), forming complex interaction networks with miRNAs; this ceRNA network serves as a core module that coordinately regulates cholesterol homeostasis in tumor cells. From a mechanistic perspective, both lncRNAs and circRNAs harbor numerous miRNA response elements (MREs), enabling them to act as molecular sponges that competitively sequester endogenous miRNAs, thereby blocking miRNA binding to their downstream target mRNAs and relieving their post-transcriptional silencing effects. For instance, miR-579-3p downregulates SQLE expression in gastric adenocarcinoma cells by directly targeting the SQLE mRNA 3’-UTR. However, studies have identified that the circRNA circ_0000182 possesses target-binding sites for miR-579-3p in gastric adenocarcinoma and functions as a ceRNA by sponging miR-579-3p, consequently promoting SQLE expression, cholesterol synthesis, and cell proliferation [62]. The lncRNA HULC was among the first oncogenic ncRNAs identified as being highly upregulated in HCC. Mechanistically, HULC induces methylation of the CpG island in the miR-9 promoter, thereby epigenetically silencing miR-9. This relieves the miR-9-mediated repression of PPARα mRNA, leading to increased PPARα expression. Upregulated PPARα, in turn, transactivates genes such as acyl-CoA synthetase long-chain family member 1, which is involved in cholesterol metabolism. Furthermore, PPARα enhances HCC cell proliferation by activating the oncogene RXRA. Intriguingly, cholesterol itself can upregulate HULC expression via an RXRA-mediated positive feedback loop, establishing a self-reinforcing oncogenic circuit [63]. Within the tumor ceRNA regulatory network, various classes of non-coding RNAs form a tandem regulatory axis from lncRNA/circRNA to miRNA and then to cholesterol metabolism genes. This synergistic regulatory pattern transcends the independent action of a single ncRNA, constituting an interconnected ceRNA regulatory system that dynamically and precisely modulates cholesterol metabolism. In summary, ncRNAs and their ceRNA networks exert precise control over cholesterol homeostasis by targeting central regulators such as SREBP2, HMGCR, LDLR, and ABCA1/ABCG1, thereby critically influencing tumor progression. These findings underscore the considerable potential of ncRNAs and their ceRNA networks to serve as both novel biomarkers and therapeutic targets. Future research should therefore focus on developing precision metabolic intervention strategies based on ncRNAs and their ceRNA networks, opening new avenues for cancer therapy.

### Mitochondrial cholesterol metabolism in cancer cells

Although cholesterol primarily regulates numerous signaling pathways at the plasma membrane, mitochondria serve as critical organelles that govern cholesterol transport and redox homeostasis. Intracellular free cholesterol is predominantly transported to mitochondria via non-vesicular trafficking, a process mediated by START domain-containing sterol transfer proteins, including STARD1, STARD3, and STARD4. Among these, STARD1 is the principal protein responsible for mediating cholesterol transport from the outer to the inner mitochondrial membrane, where it initiates subsequent metabolic reactions, whereas STARD3 and STARD4 act synergistically to facilitate cholesterol delivery to the outer mitochondrial membrane. Free cholesterol is dynamically translocated to the mitochondrial inner membrane via multiple carrier proteins, where it participates in the synthesis of steroids and oxysterols [64].

**Fig. 2:**
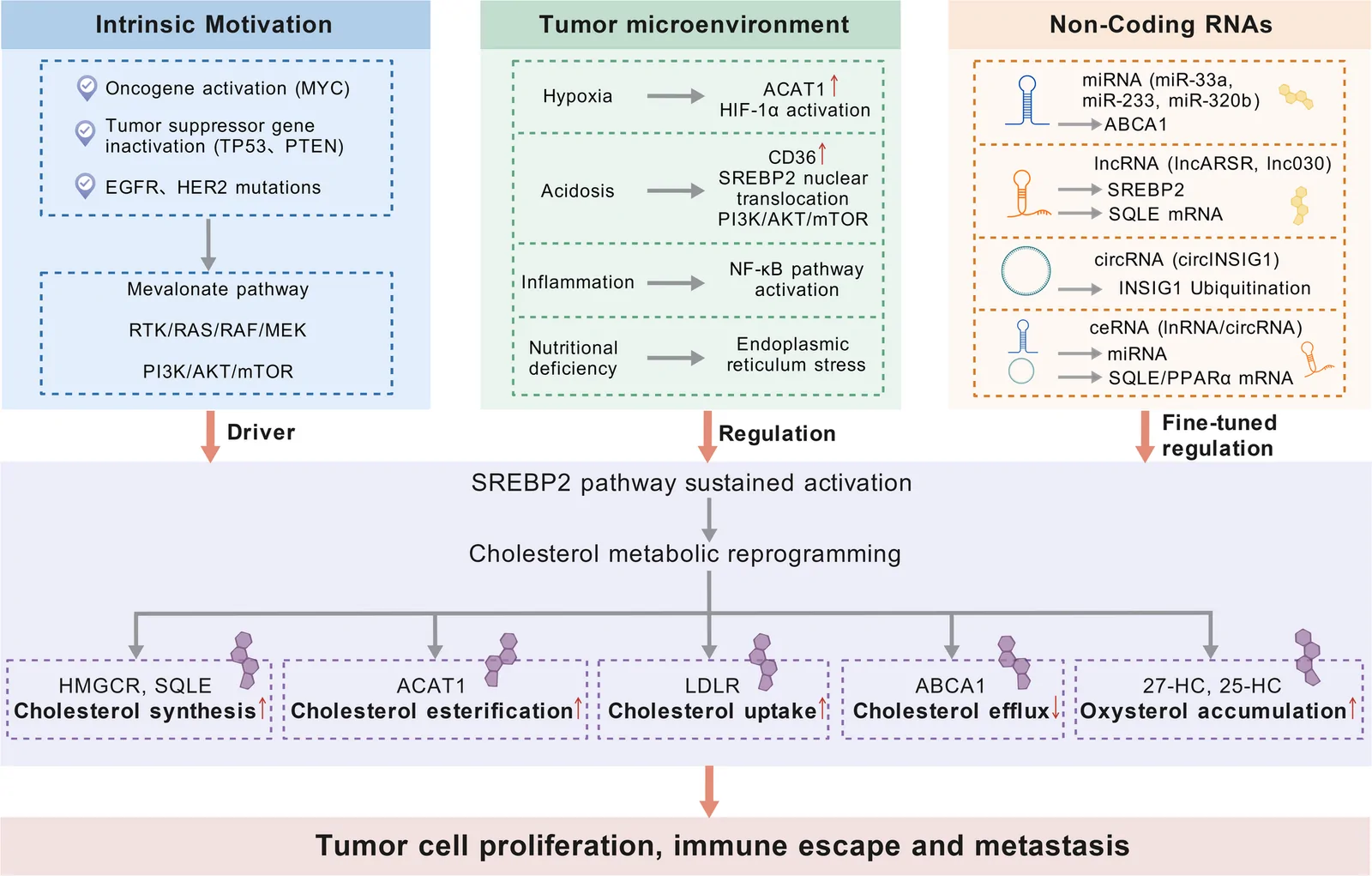
Core mechanisms of cholesterol metabolic reprogramming in tumors and its impact on malignant phenotypes. Tumor cholesterol metabolic reprogramming is orchestrated by a multi-layered regulatory network. Intrinsic genetic alterations, including activation of proto-oncogenes (e.g., MYC), inactivation of tumor suppressors (e.g., TP53, PTEN), and mutations in receptors (e.g., EGFR, HER2), serve as fundamental drivers of metabolic reprogramming. These alterations converge to hyperactivate key pathways such as the mevalonate pathway, the RAS/RAF/MEK cascade, and the PI3K/AKT/mTOR axis. Extrinsically, specific conditions within the TME persistently activate the SREBP2 pathway through distinct mechanisms, further promoting cholesterol metabolic reprogramming: (a) Hypoxia: activates HIF-1α and upregulates ACAT1, promoting cholesterol esterification; (b) Acidosis: induces CD36 upregulation, facilitates SREBP2 nuclear translocation, and activates the PI3K/AKT/mTOR pathway, thereby boosting cholesterol synthesis and uptake; (c) Inflammation: activates the NF-κB pathway, which in turn sustains the activity of metabolic reprogramming processes; (d) Nutrient deprivation: induces ER stress, which indirectly modulates the expression of key cholesterol metabolic regulators. Furthermore, non-coding RNAs fine-tune cholesterol metabolism by targeting critical nodes: (a) miRNAs (e.g., miR-33a, miR-233, miR-320b): post-transcriptionally repress ABCA1, impairing cholesterol efflux and promoting intracellular accumulation; (b) lncRNAs (e.g., lncARSR, lnc030): stabilize SQLE mRNA and modulate SREBP2 expression, thereby augmenting cholesterol synthesis; (c) circular RNAs (e.g., circINSIG1): encode peptides that promote INSIG1 ubiquitination and degradation, relieving its inhibition on cholesterol synthesis and thus boosting cholesterol production; (d) ceRNA regulatory axis: lncRNAs and circRNAs act as ceRNAs to sponge corresponding miRNAs, thereby relieving miRNA-mediated repression of target transcripts, including SQLE and PPARα mRNAs. Persistent SREBP2 pathway activation driven by the above three regulatory layers simultaneously reshapes multiple cholesterol metabolic processes, including elevated cholesterol synthesis (HMGCR, SQLE), enhanced cholesterol esterification (ACAT1), increased cholesterol uptake (LDLR), inhibited cholesterol efflux (ABCA1), and massive oxysterol accumulation (27-HC, 25-HC). Ultimately, this comprehensive cholesterol metabolic reprogramming synergistically facilitates the core malignant characteristics of tumor cells, namely proliferation, immune escape, and metastasis

Experimental evidence indicates that elevated hepatic cholesterol levels induced by a high-cholesterol diet in mice upregulate STARD1 expression and stimulate mitochondrial cholesterol delivery [65]. In cancer cells, aberrant mitochondrial cholesterol accumulation resulting from STARD1 overexpression disrupts respiratory chain complex assembly and triggers substantial bursts of mitochondrial reactive oxygen species [66]. This perturbed mitochondrial redox milieu promotes non-enzymatic oxidation of cholesterol, leading to the accumulation of pro-tumorigenic oxysterols such as 7-KC and 27-HC, thereby establishing a positive feedback loop between cholesterol accumulation and oxidative stress. Furthermore, NADH accumulation arising from tricarboxylic acid cycle dysfunction disrupts the intracellular NAD⁺/NADH balance, which in turn stabilizes nuclear SREBP2, continuously promoting *de novo* cholesterol synthesis and thereby enhancing the metabolic plasticity of tumor cells. (Fig. 2)

## The functional significance of cholesterol metabolism in cancer

### Regulation of immune responses

Cholesterol metabolism critically modulates antitumor immunity, primarily through the regulation of key immune-related signaling pathways. This immunomodulatory role represents a significant mechanism through which dysregulated cholesterol metabolism contributes to both tumor initiation and progression. The aberrant accumulation of cholesterol within tumor cells drives the upregulation of immunosuppressive checkpoint molecules. This process facilitates tumor immune escape and substantially attenuates the host’s antitumor immune response.

Elevated secretion of fibroblast growth factor 21 across multiple tumor types has been shown to activate the AKT-mTORC1 signaling pathway, which promotes cholesterol biosynthesis and ultimately drives the exhaustion of cytotoxic CD8^+^ T cells [67]. For instance, in CRC cells, the endogenous vacuolar ATPase subunit ATP6V0A1 facilitates exogenous cholesterol uptake via a pathway dependent on the RAB guanine nucleotide exchange factor 1-mediated endosomal maturation. This leads to cholesterol accumulation within the ER and enhanced production of 24-hydroxycholesterol, a potent activator of LXR signaling. Consequently, this pathway upregulates transforming growth factor-β1 expression, which suppresses CD8^+^ T cell activation and thereby facilitates immune escape in CRC [68]. These findings indicate that cholesterol metabolism can modulate the function of immune effector cells through intricate metabolic networks, disrupt immune surveillance, and ultimately subvert antitumor immunity.

Beyond its role in immune cell function, cholesterol metabolism is intrinsically linked to the stability and expression of immune checkpoint molecules, most notably the programmed cell death ligand 1 (PD-L1). Mechanistically, the transmembrane domain of PD-L1 harbors two cholesterol-binding motifs known as cholesterol recognition/interaction amino acid consensus (CRAC) motifs: CRAC1 and CRAC2. Cholesterol molecules directly bind to these CRAC motifs. This interaction stabilizes PD-L1 by shielding it from degradation, thereby promoting its accumulation on the tumor cell surface and facilitating immune evasion [69]. These mechanistic insights reveal that reducing cholesterol to diminish PD-L1 expression constitutes a novel and promising therapeutic strategy to counteract immune evasion in cancer.

The abnormal accumulation of cholesterol and its derivatives within the TME serves to recruit, enrich, and activate a spectrum of immunosuppressive cells, such as myeloid-derived suppressor cells (MDSCs), regulatory T cells (Tregs), and tumor-associated macrophages (TAMs). This process actively shapes an immunosuppressive TME and significantly attenuates antitumor immune responses. One mechanism involves the transcription factor X-box binding protein 1 in tumor cells, which directly binds to the promoter of the HMGCR gene to upregulate its expression, thereby activating *de novo* cholesterol biosynthesis. The increased cholesterol enhances STAT3 phosphorylation and elevates inducible nitric oxide synthase mRNA expression. This, in turn, stimulates the activation and expansion of MDSCs, suppresses antitumor immunity, and promotes tumor growth. An alternative pathway is influenced by systemic metabolism. The presence of cancer and a high-cholesterol diet can independently or synergistically elevate systemic cholesterol levels. This dysregulated systemic cholesterol metabolism significantly upregulates the expression of retinoic acid-related orphan receptor γ within MDSCs. The subsequent activation of the associated dependent signaling drives the expansion of both MDSCs and M2-polarized TAMs, amplifies immunosuppressive effects, and ultimately accelerates cancer progression [70].

Beyond shaping the immunosuppressive milieu, cholesterol metabolism also critically impacts antigen presentation, a cornerstone of adaptive antitumor immunity. Aberrant cholesterol accumulation impairs antigen-presenting cells (APCs), including dendritic cells (DCs), by downregulating expression of major histocompatibility complex (MHC) molecules. This suppression of MHC expression inhibits APC maturation and activation, ultimately compromising their antigen-presentation capacity [71]. Additionally, cholesterol can directly modify the formation of tumor-associated antigens, enabling tumor cells to evade immune surveillance [72]. The cGAS-STING signaling pathway is another critical target of cholesterol-mediated immunomodulation, as it plays a pivotal role in initiating innate antitumor immune responses. Mechanistically, cholesterol directly binds to the CRAC motif within STING. This interaction sequesters STING in the ER, preventing its trafficking to the Golgi apparatus and subsequent activation, thereby suppressing this key innate immune pathway and undermining antitumor immunity [73].

In summary, cholesterol metabolism in cancer primarily modulates immune responses within the TME through multiple mechanisms, including dysfunction of antitumor effector cells, expansion and hyperactivation of immunosuppressive cell populations, and impaired antigen presentation and recognition. These mechanisms converge to establish and sustain a profoundly immunosuppressive TME, which is a fundamental driver of tumor immune evasion and progression.

### Abnormal cholesterol metabolism induces ferroptosis and autophagy in cancer cells

In recent years, the crosstalk between dysregulated cholesterol metabolism and ferroptosis has emerged as a pivotal area of research in cancer biology. Ferroptosis is an iron-dependent novel form of programmed cell death characterized by the lethal accumulation of ROS and lipid peroxidation. Its core biochemical mechanism involves the peroxidation of polyunsaturated fatty acids within cellular membranes, catalyzed by ferrous iron or lipoxygenases, which ultimately leads to cell death. In cancer, dysregulated cholesterol metabolism can modulate ferroptosis through diverse molecular pathways, offering new targets for cancer therapy. For instance, sustained activation of the PI3K/AKT/mTORC1 signaling axis upregulates SREBP1, which in turn promotes the expression and activity of stearoyl-CoA desaturase-1. Stearoyl-CoA desaturase-1 is an iron-dependent enzyme that catalyzes fatty acid desaturation. By converting saturated fatty acids to monounsaturated counterparts, this reaction reduces the cellular pool of ferroptosis-susceptible lipids, thereby conferring resistance to ferroptosis in cancer cells [74]. Another link is established through 7-dehydrocholesterol (7-DHC), a cholesterol biosynthesis intermediate that functions as an endogenous inhibitor of ferroptosis. Accumulation of 7-DHC enables cancer cells to adopt a ferroptosis-resistant state, allowing them to evade this form of cell death and potentially acquire a more aggressive phenotype. Accordingly, pharmacological inhibition of 7-DHC biosynthesis can induce ferroptosis and suppress tumor growth, highlighting its therapeutic potential [75]. In HCC, transmembrane protein 147 (TMEM147) is frequently overexpressed compared to normal hepatocytes. TMEM147 enhances the transcriptional activity of STAT2, leading to upregulation of 7-DHC reductase and consequently promoting the biosynthesis of 27-HC. Given that the glutathione (GSH)-glutathione peroxidase 4 (GPX4) axis is the primary defense against ferroptosis, the finding that 27-HC upregulates GPX4 in HCC is significant. This suggests that the ferroptosis resistance conferred by the TMEM147/STAT2/7-DHC reductase axis is mediated predominantly through 27-HC, ultimately facilitating HCC proliferation and metastasis [76].

Autophagy refers to the process whereby cytoplasmic components, such as damaged organelles, long-lived proteins, and invading pathogens, are transported to lysosomes for degradation. A key step in autophagy involves the formation of multiple membrane structures, with the generation of double-membraned autophagosomes. Cholesterol metabolism is emerging as a critical regulator of autophagy. The mTORC1 is a master negative regulator of autophagy. Cholesterol activates mTORC1 by facilitating its recruitment and translocation from the cytosol to the lysosomal surface. Consequently, aberrant cholesterol accumulation inhibits autophagy via mTORC1 hyperactivation, a mechanism that can promote cancer cell survival. For instance, inhibition of LDLR-related protein 8 (LRP8), a mediator of cholesterol uptake, reduces cholesterol absorption in multiple myeloma cells. This suppression attenuates cholesterol-dependent mTORC1 activity, thereby triggering a protective autophagic response. LRP8 is closely associated with multiple myeloma patient prognosis and disease stage, underscoring its potential as a therapeutic target [77]. The GRAMD protein family is characterized by a GRAM domain that mediates interaction with phosphatidylserine on membranes. A key member, GRAMD1C, functions as a non-vesicular cholesterol transporter, shuttling cholesterol from the plasma membrane to the ER via its sterol-binding VASt domain. Studies in human osteosarcoma U2OS cells have shown that depleting plasma membrane cholesterol (e.g., using methyl-β-cyclodextrin or atorvastatin) or genetically ablating GRAMD1C both induce autophagy [78]. In summary, aberrant cholesterol metabolism in cancer impacts autophagic activity through multiple, interconnected molecular pathways.

**Fig. 3:**
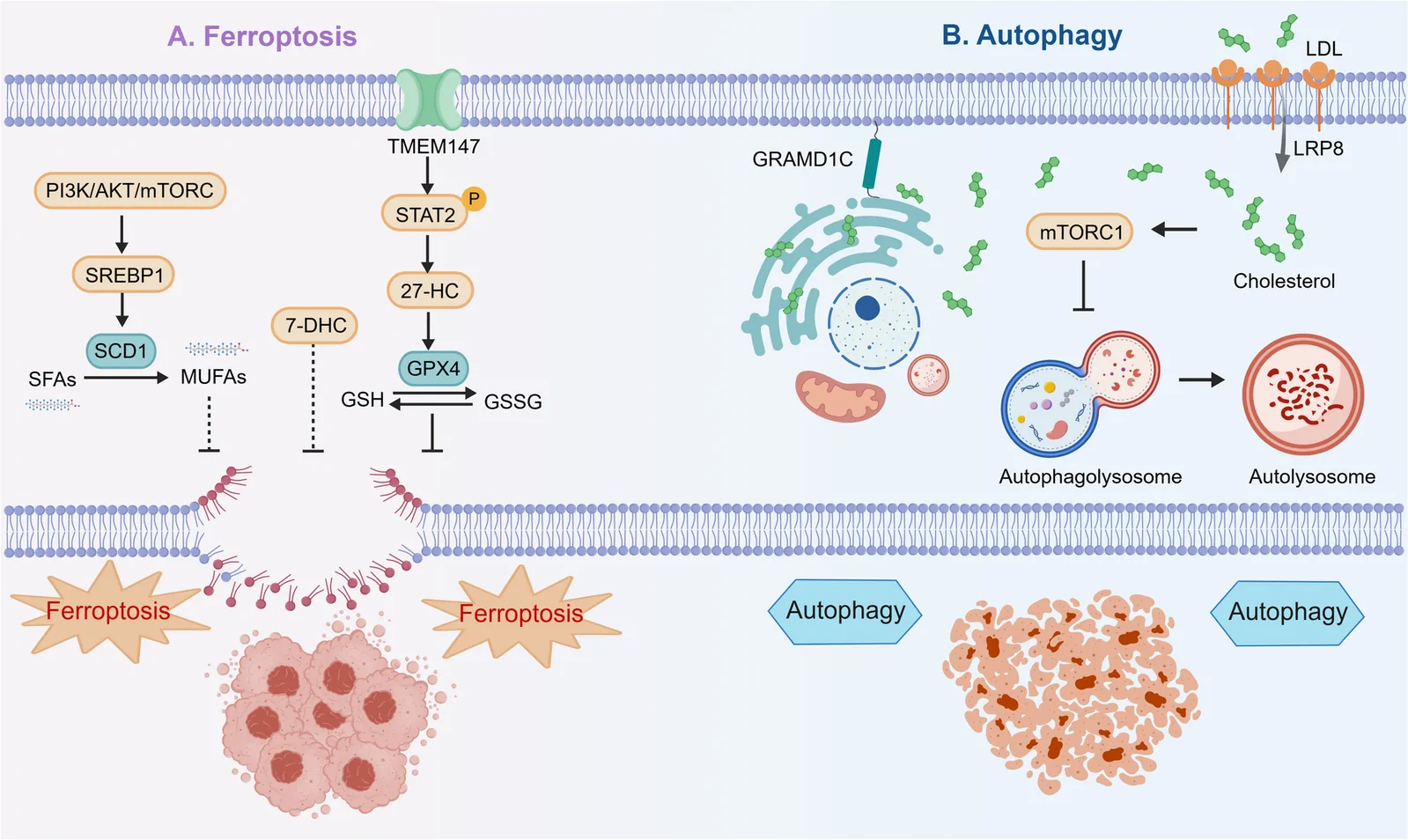
Molecular mechanisms by which aberrant cholesterol metabolism regulates ferroptosis and autophagy in tumor cells. Dysregulated cholesterol metabolism modulates the susceptibility of tumor cells to ferroptosis and alters autophagic flux through multiple signaling pathways. **A** Cholesterol metabolism regulates the ferroptosis pathway: Sustained activation of the PI3K/AKT/mTORC1 axis upregulates SREBP1, which in turn activates SCD1, promoting the desaturation of saturated fatty acids to monounsaturated fatty acids and thereby conferring ferroptosis resistance to tumor cells. Concurrently, the cholesterol synthesis intermediate 7-DHC acts as an endogenous suppressor of ferroptosis, and its accumulation enables tumor cells to evade ferroptotic cell death. Additionally, the membrane protein TMEM147 mediates STAT2 phosphorylation, which in turn drives the production of 27-HC. 27-HC further upregulates GPX4 expression, thereby enhancing the GSH-GPX4 antioxidant axis to attenuate lipid peroxidation and mediate ferroptosis resistance in cancer cells. **B** Cholesterol metabolism regulates the autophagy pathway: The cell membrane receptor LRP8 and the non-vesicular cholesterol transporter GRAMD1C influence cholesterol uptake and transport. mTORC1 serves as a core negative regulator of autophagy; intracellular cholesterol accumulation promotes mTORC1 translocation to the lysosomal membrane and sustains its activation. Consequently, hyperactivated mTORC1 blocks autophagosome maturation into autolysosomes, globally suppresses autophagic flux, and ultimately promotes tumor cell survival

Inhibition of mitochondrial carriers such as SLC25A10 and SLC25A11 is accompanied by elevated mitochondrial ROS, membrane depolarization, and GSH depletion, thereby inducing ferroptosis in cardiomyocytes [79]. In cancer cells, aberrant mitochondrial cholesterol accumulation reduces membrane fluidity and increases membrane order, which further specifically impairs mitochondrial carrier function and disrupts mitochondrial GSH transport. Increased mitochondrial cholesterol loading in mice fed a high-cholesterol diet reduces state 3 respiration driven by Complexes I and II, diminishes mitochondrial membrane potential and the respiratory control ratio of Complex I, and impairs respiratory chain supercomplex assembly [80, 81]. These findings indicate that cholesterol directly impacts oxidative phosphorylation and consequently promotes oxidative stress [65]. Furthermore, mitochondrial cholesterol accumulation suppresses mitochondrial outer membrane permeabilization, thereby impairing cytochrome c release and subsequent apoptosome assembly [82]. Converging evidence indicates that elevated mitochondrial cholesterol levels upregulate the mitophagy regulator PTEN-induced kinase 1 and the autophagy marker LC3II, thereby promoting mitophagosome formation [83–85]. In clear cell renal cell carcinoma (ccRCC), SLC27A3 overexpression catalyzes long-chain fatty acyl-CoA generation, accompanied by increased lipid synthesis and ROS production, and prevents PTEN-induced kinase 1 degradation to activate mitophagy, thereby mediating Pazopanib resistance [86]. Collectively, mitochondrial GSH depletion and elevated ROS resulting from cholesterol accumulation impair respiratory chain supercomplex assembly and oxidative phosphorylation [87], suppress mitochondria-mediated apoptotic pathways, severely compromise mitochondrial function and autophagic flux, and ultimately precipitate chemotherapy resistance in tumors. (Fig. 3).

### Tumor cell stemness

Cancer stem cells (CSCs), defined by their capacities for self-renewal, differentiation, and therapy resistance, are widely regarded as the root cause of tumor metastasis, recurrence, and therapeutic failure. Dysregulated cholesterol metabolism has been implicated in promoting tumor relapse and metastasis across various cancers, primarily through the modulation of CSC stemness. For example, in breast cancer, both SQLE overexpression and consequent cholesterol accumulation have been shown to upregulate levels of phosphorylated PI3K and AKT. This activation of the PI3K/AKT pathway potently enhances the stemness properties of breast CSCs. In CRC, upregulation of cholesterol synthesis genes, including HMGCR, activates the downstream Sonic hedgehog signaling pathway. This activation subsequently promotes CRC cell proliferation and augments stemness [88]. In summary, these findings suggest that pharmacological targeting of cholesterol metabolism represents a promising strategy to attenuate CSC stemness, thereby overcoming therapy resistance and preventing tumor recurrence.

### DNA damage response

DNA damage refers to abnormal alterations in the cellular genome, which may be induced by endogenous factors (e.g., ROS) or exogenous factors (e.g., radiation, chemical mutagens). Under physiological conditions, cells activate the DNA damage response (DDR), a sophisticated signaling network, to repair such lesions and maintain genomic integrity [89]. DNA damage and resultant genomic instability are established hallmarks of cancer. Dysregulated cholesterol metabolism can interfere with the DDR via mechanisms including oxidative stress and the accumulation of toxic metabolites. This interference exacerbates genomic instability in tumors and can contribute to resistance against anticancer therapies. For instance, lovastatin, which targets the mevalonate pathway to suppress cholesterol biosynthesis, has been shown to inhibit the activation of key DDR mediators such as checkpoint kinase 1, checkpoint kinase 2, and phosphorylated histone H2AX in gallbladder cancer cells. This impairment of the DDR sensitizes the cancer cells to cisplatin-induced apoptosis [90]. Beyond direct signaling effects, dysregulated cholesterol metabolism may also compromise DNA repair capacity indirectly by altering cell membrane fluidity/integrity and modulating the activity of associated signaling pathways. However, the precise molecular mechanisms governing the interplay between cholesterol metabolism and the DDR across diverse cancer types remain incompletely understood and warrant further investigation.

## Regulation of antitumor activity of major immune cells in the tumor microenvironment by cholesterol metabolism

The TME is a complex and dynamic ecosystem shaped by the continuous interplay among malignant cells, diverse immune cell populations, and stromal components. Among these constituents, tumor-infiltrating immune cells, such as T lymphocytes, TAMs, DCs, MDSCs, neutrophils, and natural killer (NK) cells, serve as critical regulators of tumor progression and antitumor immunity. Analysis of the intratumoral cholesterol landscape reveals that most antitumor lymphocytes exhibit cholesterol deficiency, whereas tumor cells and immunosuppressive myeloid cells display elevated cholesterol levels [26]. Numerous studies have demonstrated that cholesterol metabolism modulates tumor immune recognition and escape mechanisms, thereby attenuating antitumor immune responses [67, 91, 92].

Notably, mitochondrial metabolism and redox homeostasis also influence cholesterol-mediated remodeling of immune cell function. Perturbations in cholesterol transport to immune cell mitochondria modulate mitochondrial function and ROS production, shift the intracellular NAD⁺/NADH ratio, and regulate transcription factors such as LXR and SREBP, thereby inducing the expression of CCL2, IL-6, and IL-8. These changes subsequently promote macrophage infiltration and immunosuppressive polarization, ultimately compromising the function of cytotoxic T cells [93].

### Regulatory T cell

Tregs potentiate the immunosuppressive state of the TME primarily through the secretion of inhibitory cytokines and the expression of surface inhibitory receptors [94–96]. Tregs exhibit a high dependency on cholesterol metabolism to maintain their stability and suppressive function. This metabolic adaptation amplifies their immunosuppressive capacity, thereby promoting tumor immune escape. Mechanistic studies reveal that the activity of the SCAP-SREBP axis is markedly upregulated in Tregs [97]. This upregulation facilitates the functional maturation of Tregs by coordinating signaling through key inhibitory receptors. Furthermore, SREBP activation promotes PD-1 expression in Tregs through protein geranylgeranylation, a post-translational modification driven by mevalonate metabolism. Intriguingly, immunosuppressive TAMs rely on SREBP1-driven fatty acid synthesis, which is blocked by CD8⁺ T cell-derived interferon-γ (IFN-γ). Tregs can counteract this blockade, thereby preserving this metabolic pathway. In murine models of CRC, GBM, and melanoma, Tregs upregulate the fatty acid translocase CD36, thereby enhancing their uptake of cholesterol and other lipids. The resulting intracellular lipid accumulation activates the PPAR signaling pathway. PPAR activation further amplifies Treg immunosuppressive function by reprogramming mitochondrial metabolism [97–99]. Thus, a complex and bidirectional regulatory relationship exists between cholesterol metabolism and Tregs within the TME, collectively reinforcing an immunosuppressive microenvironment.

### T cell

Cholesterol is a critical component of lipid rafts essential for T cell receptor (TCR) signal transduction. When SREBP and LDLR expression are upregulated, the cholesterol level in both the entire cell and the plasma membrane increases, thereby promoting the formation of lipid raft structures. Furthermore, cholesterol directly binds to the transmembrane domain of the TCR-β chain, facilitating TCR clustering on the T cell surface and enhancing TCR recognition capacity for tumor antigens [100–102]. ACAT1 inhibits TCR clustering and downstream signal transduction, thereby impairing the proliferation and effector function of CD8⁺ T cells. Studies in murine melanoma models have shown that the ACAT1 inhibitor avasimibe exhibits significant antitumor effects. Combination therapy with anti-PD-1 agents further enhances therapeutic efficacy [103]. In the TME, oxidized cholesterol mediates the activity of SREBP2/LXR in T cells, leading to the downregulation of cholesterol levels. Cholesterol deprivation adversely affects the proliferation and survival of CD8⁺ cytotoxic T cells: low cholesterol levels inhibit T cell proliferation, induce T cell dysfunction, and trigger autophagy-mediated apoptosis [26]. Additionally, the binding of immune checkpoint receptors on T cells to their cognate ligands drive endogenous T cell exhaustion, suppress their tumor-killing capacity, and ultimately facilitate tumor immune escape [104, 105]. CD36 is a scavenger receptor responsible for transporting oxidized lipids and fatty acids, such as oxidized low-density lipoprotein. Studies indicate that elevated cholesterol levels in the TME upregulate CD36 expression in tumor-infiltrating CD8⁺ T cells, inducing lipid peroxidation and ferroptosis, thereby impairing their effector functions [106]. Targeting cholesterol metabolism pathways may thus enhance the antitumor function of T cells, offering novel strategies for cancer immunotherapy.

### TAM

The traditional dichotomy classifies TAMs into classically activated (M1) and alternatively activated (M2) macrophages. M1-type macrophages promote anti-tumor inflammatory responses, while M2-type macrophages exert immunosuppressive effects. Based on spatial transcriptomics and immunohistochemical analyses, multiple TAM subpopulations with distinct molecular characteristics have been identified. These findings reveal the complexity and functional diversity of TAMs, suggesting that they should be regarded as a continuous state spectrum rather than a rigid binary classification [107–110]. In the TME, metabolic reprogramming of TAMs is a core mechanism driving their transition into a pro-tumorigenic and metastasis-promoting role. In ovarian cancer, for example, tumor cell-derived hyaluronic acid (HA), which is an extracellular matrix component that promotes cholesterol efflux from TAM membranes. This leads to loss of lipid rafts and disrupts cholesterol metabolism, ultimately suppressing IFN-γ signaling and IFN-γ-induced gene expression. Consequently, these changes drive M2 polarization of TAMs and facilitate tumor growth [10, 111]. Studies have revealed that CYP27A1 expression is significantly higher in M2-polarized TAMs compared to the M1 phenotype. 27-HC activates M2-type TAMs, thereby promoting tumor cell proliferation. Additionally, 27-HC stimulates TAMs to secrete chemokines (e.g., CCL2 and CCL3), which recruit monocytes to tumor sites and facilitate tumor progression [112]. In summary, cholesterol metabolic reprogramming in TAMs critically influences their functional polarization. Targeting cholesterol metabolism pathways to reverse the pro-tumorigenic phenotype of TAMs may represent a novel therapeutic strategy for cancer treatment.

Beyond influencing polarization states, cholesterol metabolism critically regulates the immunometabolic plasticity of TAMs by reprogramming mitochondrial function and redox adaptation. In GBM, cholesterol accumulation has been shown to impair mitochondrial translation in TAMs, thereby compromising macrophage phagocytosis through mitochondrial dysfunction. Promoting cholesterol efflux with apolipoprotein A1 mediates lipid-associated metabolic remodeling and reduces 7-KC levels, directly alleviating mitochondrial translational impairment. This, in turn, suppresses tumor necrosis factor signaling in TAMs, restores TAM phagocytic capacity, and reactivates TAM-T cell antitumor immunity [113]. Furthermore, aberrant cholesterol accumulation within TAM mitochondria impairs mitochondrial respiratory function, disrupts cellular redox homeostasis, and exacerbates ROS production, thereby perpetuating the immunosuppressive state of the TME. Concurrently, excessive ROS production leads to the release of mitochondrial DNA into the cytoplasm, triggering the secretion of protumorigenic chemokines such as CCL2 and CCL3 as well as immunosuppressive cytokines (e.g., IL-10 and TGF-β) and activating the NLRP3 inflammasome via the cGAS-STING pathway, which subsequently promotes macrophage infiltration and immunosuppressive polarization. Additionally, this process upregulates the expression of multiple immune checkpoint molecules, including PD-L1, on the TAM surface, and these mechanisms synergistically suppress antitumor immune responses [93, 114].

### DC

DCs, a type of APC, present exogenous antigens via MHC-I and MHC-II molecules to effector T cells, thereby activating their cytotoxic functions against tumor cells. Apolipoprotein E (APOE), encoded by the APOE gene, serves as a critical carrier for cholesterol and lipids. Deficiency in APOE leads to cholesterol accumulation on DC membranes, resulting in clustering of MHC-II molecules on the cell surface. This enhances antigen presentation to T cells and initiates adaptive immune responses [115]. However, cholesterol can also suppress the tumor antigen presentation function of APCs. Glycosphingolipids, which are tumor-associated antigens overexpressed on tumor cells and CSCs, can bind to cholesterol. This interaction alters the conformation of glycosphingolipids, rendering them unrecognizable by APCs and thereby inhibiting antitumor immune responses [72]. Additionally, oxysterols activate LXRα to suppress CC chemokine receptor 7 (CCR7) expression. CCR7 is a critical receptor for DC migration to lymph nodes; its downregulation reduces MHC-I expression on DC surfaces and impairs DC trafficking to lymph nodes, ultimately weakening antigen presentation [116]. Furthermore, in HCC cells, alpha-fetoprotein (AFP) produced downregulates SREBP expression in DCs, leading to decreased synthesis of cholesterol and other lipids. This compromises their ability to activate T cells and promotes HCC progression [117].

### NK cell

NK cells are critical components of the innate immune system and can directly kill tumor cells. Research has demonstrated that excessive intracellular cholesterol accumulation in NK cells exerts detrimental effects on their activity. Specifically, intracellular cholesterol disrupts the formation of membrane lipid rafts, compromising receptor localization and the activation of NK cell signaling pathways. Furthermore, elevated cholesterol levels downregulate the expression of perforin, IFN-γ, and TNF-α, thereby impairing the cytotoxic capacity of NK cells [118]. Conversely, activated NK cells can further upregulate SREBP and HMGCR, enhancing cholesterol biosynthesis and thereby amplifying their antitumor activity [119]. These findings demonstrate that NK cell activation signaling relies on lipid raft formation. Both cholesterol deficiency and excess, representing aberrant cholesterol metabolism, can disrupt lipid rafts, consequently suppressing NK cell cytotoxicity.

### Neutrophil

**Fig. 4:**
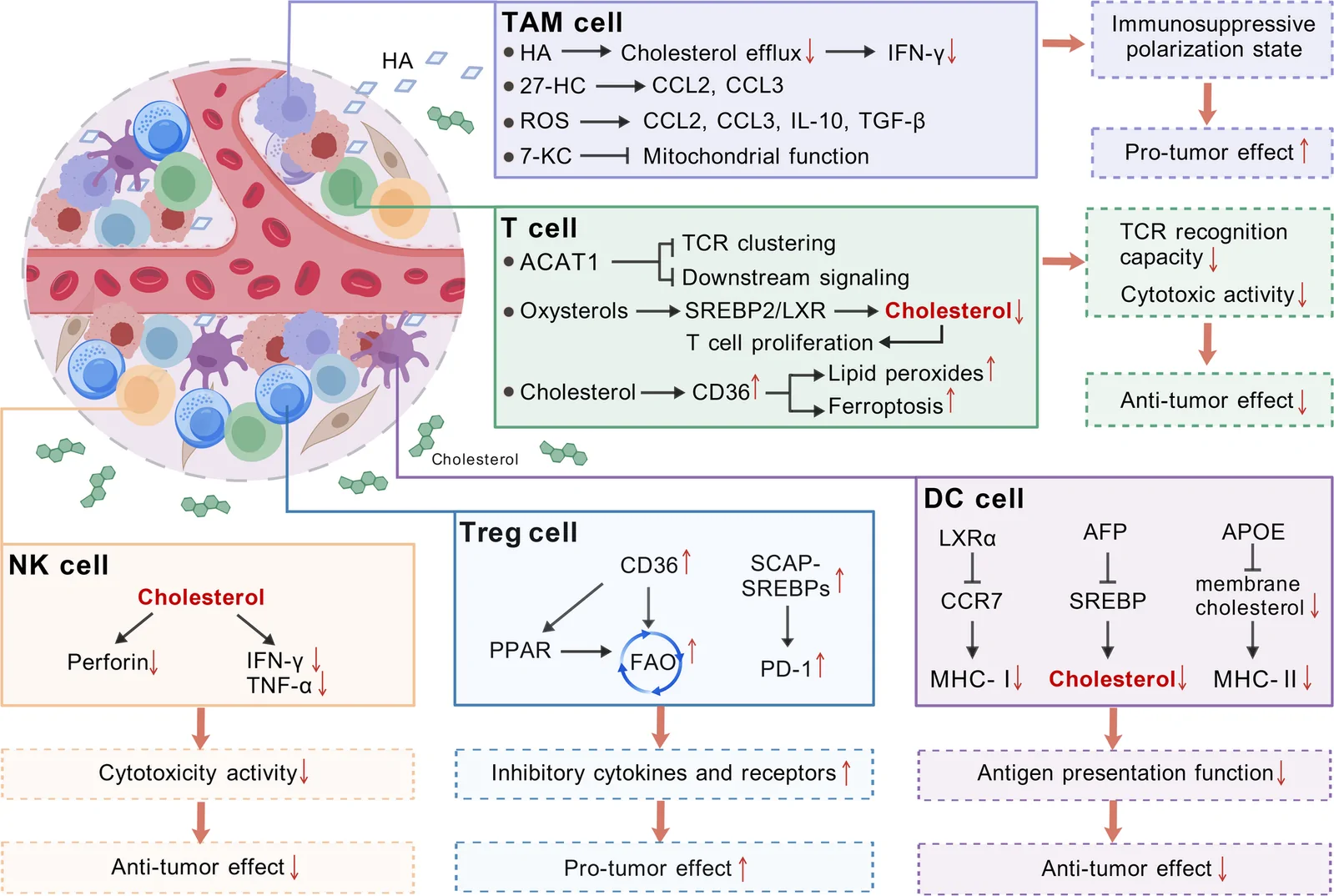
Regulation of cholesterol metabolism in different immune cells and its impact on tumor progression. In TAMs, HA increases cholesterol efflux, thereby suppressing IFN-γ secretion and promoting M2 polarization. The cholesterol metabolite 27-HC, in synergy with ROS, induces TAMs to secrete the protumorigenic chemokines CCL2 and CCL3. In addition, ROS triggers the secretion of immunosuppressive cytokines, such as IL-10 and TGF-β. Meanwhile, 7-KC impairs TAM mitochondrial function, consequently attenuating their phagocytic capacity. Collectively, these mechanisms enable TAMs to maintain a stable immunosuppressive polarization phenotype and synergistically exert pro-tumorigenic effects. In T cells, multiple disturbances in cholesterol metabolism impair T cell function through distinct mechanisms. ACAT1 inhibits TCR clustering and downstream signal transduction. Oxysterols modulate the SREBP2/LXR pathway to reduce intracellular cholesterol levels and suppress T cell proliferation. Moreover, cholesterol accumulation upregulates CD36 expression, leading to lipid peroxide accumulation and increased ferroptosis. Collectively, these metabolic abnormalities result in diminished TCR antigen recognition capacity and reduced cytotoxic activity, ultimately leading to a marked attenuation of T cell-mediated antitumor immunity. In DCs, LXRα activation suppresses CCR7 expression, impedes DC migration to lymph nodes, and reduces MHC-I expression. AFP downregulates SREBP expression, thereby impairing cholesterol synthesis and T cell activation capacity. Meanwhile, APOE deficiency leads to cholesterol accumulation and MHC-II clustering, which in turn inhibits antigen-presenting function and antitumor efficacy. Additionally, in Tregs, upregulated CD36 expression promotes the uptake of cholesterol and other lipids, concomitantly activating PPAR and FAO. Moreover, the SCAP-SREBPs cascade is markedly upregulated in Tregs, promoting PD-1 expression. Consequently, cholesterol metabolic reprogramming increases the secretion of inhibitory cytokines and enhances the immunosuppressive function of Tregs, thereby exerting pro-tumorigenic effects. Furthermore, in NK cells, excessive intracellular cholesterol significantly downregulates the expression of the effector molecules perforin, IFN-γ, and TNF-α, directly attenuating NK cell-mediated cytotoxicity and antitumor capacity

In the TME, neutrophils typically exhibit pro-tumorigenic activities. Oxysterols bind to and activate the G protein-coupled receptor C-X-C motif chemokine receptor 2, thereby recruiting neutrophils, promoting angiogenesis, and enhancing immunosuppressive effects [120]. Additionally, hypoxia-induced HIF-1α upregulates CYP46A1 expression, stimulating cholesterol esterification and converting cholesterol into the oxysterol 24 S-hydroxycholesterol. Studies have demonstrated that in pancreatic neuroendocrine tumors, 24 S-hydroxycholesterol recruits pro-angiogenic neutrophils, facilitating malignant progression [121]. (Fig. 4).

## Cholesterol-lowering therapy

In summary, cholesterol metabolic reprogramming is closely associated with the initiation and progression of various cancers. In recent years, targeting cholesterol metabolism pathways has garnered significant attention for cancer therapy. However, the efficacy of cholesterol-lowering therapy varies substantially across different tumor types. Moreover, the interaction between cholesterol metabolism and the TME is complex and dynamic. Future research should further explore the mechanisms and clinical applications of cholesterol-lowering strategies in oncology.

### Targeting cholesterol synthesis

Many basic studies have shown the potential therapeutic efficacy of targeting cholesterol metabolism in cancer therapy, such as by inhibiting the mevalonate pathway in the cholesterol synthesis pathway, has been proven to be a viable anti-tumor strategy [122–124].

To date, statins, which are HMGCR inhibitors, have been used extensively in clinical studies for cancer patients via targeting cholesterol metabolism. Recent studies have reported a 31–37% reduction in mortality hazard ratio of statin users in lung cancer treatment and showed the survival benefit of statin exposure in lung cancer patients with lung-cancer-associated pleural effusion [125]. Simvastatin, an inhibitor of the mevalonate pathway, synergizes with anti-PD-L1 antibodies by driving inflammasome-regulated immunomodulatory pyroptosis [126]. Simvastatin increased the recruitment of CD8^+^ T cells into tumor sites and the production of type I IFN chemokines (CCL3, CXCL9, CXCL10, and CXCL11), augmented PD-1 blockade therapy in microsatellite stable immune-cold gastric cancer [127]. Statin treatment decreased the expression of Wnt target genes, such as anti-apoptotic protein survivin, by a Rac1/β-catenin axis, to inhibit APC-mutated CRC [128]. The pharmacological classification of statins was grouped into hydrophilic statins and lipophilic statins based on their lipophilicity and hydrophilicity. Simvastatin, fluvastatin and pitavastatin are classified as lipophilic statins, where a pravastatin and rosuvastatin belong to hydrophilic statins. Some researches indicated that only lipophilic statins not hydrophilic statins were effective to decrease the risk cancer recurrence and mortality [129].

SQLE, a rate-limiting enzyme in cholesterol synthesis, is also closely related to the development and prognosis of some tumors. Studies have shown that overexpression of SQLE is associated with a poor prognosis for human HCC. SQLE promotes the development of metabolic dysfunction-associated steatohepatitis-associated HCC by attenuating the function of Granzyme B^+^ and IFN-γ^+^ CD8^+^ T cells and increasing the number of arginase-1^+^ (Arg-1^+^) MDSCs. Terbinafine, a highly selective and effective inhibitor targeting SQLE, rescued the efficacy of anti-PD-1 immunotherapy for metabolic dysfunction-associated steatohepatitis-associated HCC in mouse models with humanized immune system [130]. 2, 3-oxidosqualene cyclase (OSC) catalyzes the conversion of 2, 3-monoepoxysqualene to lanosterol, which is a key element in the process of cholesterol biosynthesis. RO 48-8071, a selective inhibitor of OSC, inhibited the JNK and ERK/MAPK axis, further triggered cell cycle arrest by modulating cyclin B1, cyclin E and p27 expression to block pancreatic cancer growth. These findings uncovered a crucial function of OSC in the regulation of cancer progression and Ro 48-8071 as a potential anti-tumor drug [131]. Squalene synthase mediates the reductive dimerization of FPP to produce squalene in the first step of cholesterol synthesis. Zaragozic acid A belongs to a fungal metabolite that plays a reversible inhibitory effect on squalene synthase. Zaragozic acid A upregulates endogenous geranylgeranoic acid levels to induce cell death in human hepatoma cells via TLR4-mediated pyroptosis in the spontaneous hepatoma mice model [132].

Fatostatin was used as an inhibitor of SREBP2 and decreased SREBP2-mediated cholesterol aggregation and X-box binding protein 1-dependent ER stress, restraining Tregs and mitigating CD8^+^ T cell exhaustion, inhibiting tumor growth in MC38 colon cancer, and Lewis lung cancer (LLC) and B16 melanoma mice models [133].

### Targeting cholesterol uptake

The lysosomal cholesterol transporter Niemann-Pick type C1(NPC1) is overexpressed in triple-negative breast cancer (TNBC), NPC1 knockdown caused the cholesterol-filled lysosomes accumulation, and decreased aggressive characteristics in TNBC. U18666A, a well-known NPC1 inhibitor, inhibited TNBC growth and sensitized with the chemotherapeutic drug paclitaxel [134]. Glutamine is vital for tumor progression and its deprivation induced lipophagy to convey cholesterol from lipid droplets (LDs) to ER, thereby suppressing SREBF2 maturation and cholesterol synthesis. U18666A further augmented glutamine deprivation-induced block of xenograft tumor growth [135].

Itraconazole is an antifungal ergosterol synthesis drug that exerts pleiotropic capabilities such as cholesterol inhibition and mTOR pathways. Itraconazole was reported to blunt intracellular cholesterol by targeting NPC1. Itraconazole had antitumor activity by triggering lysosomal dysfunction in epithelial ovarian cancer cell lines [136]. However, itraconazole facilitated tumor-infiltrating MDSC activation to aggravate tumor growth via inhibiting the RIPK3/AKT/mTORC1 axis and induces cholesterol abrogation. It indicated that aiming at cholesterol uptake may present a therapeutic strategy to break through tumor immune evasion [137]. NPC1L1 is a cholesterol transporter, The research showed ectopically overexpressing NPC1L1 in pancreatic adenocarcinoma cells impeded TCR activation of CD8^+^ T cells and hijacked the intracellular cholesterol from CD8^+^ T cells, resulting in solving cholesterol shortage and immune surveillance of cancer cells. Ezetimibe, an NPC1L1 inhibitor, effectively prevented pancreatic adenocarcinoma cells from depriving cholesterol of CD8^+^ T cells, and improved the anti-tumor immunity activity [138].

Cholesteryl ester transfer protein (CETP) was associated with risk for breast cancer. CETP knock-down could decrease tamoxifen resistance via blockade of cholesterol accumulation in breast cancer cells, thereby inhibiting cancer aggressiveness [139]. Similarly, torcetrapib, a selective potent CETP inhibitor. sensitized ER-positive breast cancer cells to intrinsic mitochondrial apoptosis and caused less resistance towards cytotoxic compounds [140].

LDLR, identified as a cholesterol transporter, interacts with the T-cell receptor complex and promotes TCR recycling and signaling, enhancing the effector function of cytotoxic T-lymphocytes (CTLs). Proprotein convertase subtilisin/kexin type 9 (PCSK9) derived from tumor cells attenuated the effector function of CTLs via binding to LDLR and impede its function. PF-06446846, a chemical inhibitor of PCSK9, increased the antitumor activity of CTLs and prevented tumor progression [141]. Treatment with evolocumab, the PCSK9 binding antibody, impaired breast cancer metastasis in multiple models via blocking the binding of PCSK9 and tumoral LDLR.

### Targeting cholesterol efflux

Liver-X receptors (LXRα and LXRβ) are members of the nuclear hormone receptor family of transcription factors that trigger the activation of its transcriptional target APOE, associated with cholesterol metabolism. The LXR agonist RGX-104, which acts preferentially on LXRβ, activates the LXR/APOE signaling to impede the viability of MDSCs, ameliorating immunosuppression to eliminate tumor lesions [142, 143]. A selective LXR inhibitor, SR9243 downregulated the intracellular cholesterol contents, inhibited ccRCC cells proliferation but did not have cytotoxic effects on normal renal tubular epithelial cells [144]. Anti-CD133 mAb-targeted immunoliposomes encapsulating SR9243 have been successfully delivered into CD133^+^ CSCs and induced apoptosis in CRC cells [145]. LXR-623, another LXR agonist, was shown to inhibit the viability of high-grade glioma cell lines in a cholesterol-independent manner [146]. 2-hydroxypropyl-β-cyclodextrin (HP-β-CD) is a widely used drug delivery carrier. Interestingly, the study showed that HP-β-CD downregulated LXR expression and upregulated HMGCR content in breast cancer, which modulated cholesterol metabolism in association with its concentration. High level of HP-β-CD (5 mmol/L) caused cholesterol deposition in the ER, triggering ER stress, which directly Inhibited TGF-β signaling-induced epithelial-mesenchymal transition to exert anticancer effect. However, low level of HP-β-CD (0.6 and 0.8 mmol/L) exhausted membrane cholesterol and aggravate epithelial-mesenchymal transition by promoting TGF-β axis [147].

### Targeting cholesterol esterification

Avasimibe, an orally active ACAT inhibitor, attenuates tumor proliferation and metastasis by targeting E2F-1 pathway in prostate cancer [148]. In bladder cancer, avasimibe induced G1-phase cell-cycle arrest via activating PPARγ pathway and inhibited tumorigenesis [149]. Additionally, bitter melon extract treatment reduced tumor growth in TNBC mammospheres-bearing NSG mice b inhibiting ACAT1 expression [150].

### Combination strategies

The utilization of statins was correlated with breast cancer risk reduction, in addition, statins inhibited paclitaxel-triggered PD-L1 expression and promoted CD8^+^ T cytotoxicity for better prognosis in breast cancer [151]. Statin significantly reversed the immuno-cold phenotype to the inflamed phenotype in TME by inhibiting cholesterol synthesis, thereby drastically improved immune checkpoint blockade (ICB) efficacy [152]. Trastuzumab emtansine is an antibody-drug conjugate (ADC) that combines the HER2-targeted antitumor characteristics of the trastuzumab antibody with the cytotoxic activity of the microtubule-inhibitor DM1. Lovastatin enhanced HER2 membrane density in HER2^+^ gastric cancer. Use of trastuzumab emtansine together with lovastatin significantly decreased HER2 phosphorylation and HER2 protein levels, exerting synergistic therapeutic effect on tumor growth [153]. A portion of advanced prostate cancer (PCa) frequently progressed to recurrent castrate-resistant PCa in bone undergoing androgen deprivation therapy, partly due to androgens restrained cholesterol biosynthesis pathway. Statins inhibited the transition of bone marrow stromal cells to adipocytes and castration-triggered bone marrow adiposity. Statin pre-treatment reduced 22RV1 PCa progression in bone after androgen deprivation therapy. Atorvastatin effectively controlled the progression to recurrent PCa in patients undergoing androgen deprivation therapy [154]. CD8^+^ T cells have a key role in antitumor immunity, while their activity is impeded in the TME. Reactivating the function of CD8^+^ T cells is of great clinical interest in cancer immunotherapy. Cholesterol esterification inhibition caused potentiated effector function and elevated viability of CD8^+^ but not CD4^+^ T cells. The combined therapy of the anti-PD-1 antibody and avasimibe played better efficacy than monotherapies in combating tumor progression [103]. In addition, administration of Kras vaccine and avasimibe significantly upregulated intracellular granzyme B and IFN-γ levels in tumor-infiltrating CD8^+^ T cells and specifically downregulated the number of Tregs in the TME, dramatically reduced lung cancer development and growth [155]. CD8^+^ T cells activation often led to the upregulation of ACAT1 and promoted cholesterol esterification. The combination of avasimibe with chemo-immunotherapy significantly increased free cholesterol levels and alleviated CD8^+^ T cell suppression induced by Paclitaxel and immunoadjuvant αGC were co-encapsulated in liposomes modified with pH sensitive TH peptide (PTX/αGC-TH-Lip), showing enhanced chemo-immunotherapy against melanoma [156]. The combination of 5-FU and avasimibe produced a stronger synergistic effect on tumor growth of the human CRC model [157]. Bezafibrate activates the PPARα/PGC-1α signaling pathway to upregulate the expression of CPT1A, a key enzyme mediating PD-L1 succinylation, thereby promoting PD-L1 succinylation and subsequent degradation. The combination therapy of Bezafibrate with anti-CTLA-4 monoclonal antibody significantly prolongs survival in melanoma models without inducing significant systemic toxicity such as body weight loss [158].

Endocrine therapy belongs to the mainstay of hormone-receptor positive (HR^+^) breast cancer treatment, while about 40% HR^+^ breast cancer died of recurrences and progression due to inevitable endocrine resistance. The mevalonate pathway was through the downstream activation of signaling pathways including RAS/MEK/ERK and PI3K/AKT/mTOR to affect cancer cell survival, was closely related with endocrine resistance in HR^+^ breast cancer. Statins inhibit the mevalonate pathway, thereby preventing it from disrupting downstream signaling axes involved in estrogen receptor expression and key cellular functions such as cell survival, and restraining the progression of endocrine therapy resistance [159].

Metformin is the classic first-line of treatment for Type 2 diabetes. Some studies showed that administration with metformin and pitavastatin had a synergistic anticancer function on pancreatic cancer cells, because of the inhibition of PI3K/mTOR pathway, HMGCR and activation of AMP-activated protein kinase (AMPK) [160]. Docetaxel, a standard of chemotherapy regimen in metastatic castration-resistant prostate cancer (mCRPC) treatment, while mCRPC remains incurable, both CSCs and Yes-associated protein activation have been closely linked to chemo-resistance. The AMPK activator valproic acid combined simvastatin increased the susceptibilities of mCRPC to docetaxel by inhibiting Yes-associated protein and targeting CSC through concurrent regulation of AMPK and mevalonate pathway [161]. (Table 1).

**Table 1:** Clinical progress of cholesterol-lowering metabolism drugs in cancer therapy

Strategies	Drug	Target	Cancer type	Mechanism
Targeting cholesterol synthesis	Statins	HMGCR	Ovarian carcinoma, HCC, breast cancer, CRC, lung cancer	Boosted antitumor immunity, suppressed tumor cell growth and improved prognosis [28, 125–129, 151]
	Ro48-8071	OSC	Pancreatic cancer	Deactivated the JNK and ERK/MAPK signaling pathway [131]
	Terbinafine	SQLE	HCC	Inhibited SQLE and synergized with anti-PD-1 therapy [130]
	Zaragozic acid A	Squalene synthase	HCC	Upregulated hepatic geranylgeranoic acid to induce cell death in human hepatoma cells via TLR4-mediated pyroptosis [132]
	Fatostatin	SREBP2	Melanoma, colon cancer, Lewis lung cancer	Decreased SREBP2-mediated cholesterol accumulation and ER stress, restraining Tregs and mitigating CD8^+^ T cell exhaustion [133]
Targeting cholesterol uptake	U18666A	NPC1	TNBC, HCC	Decreased ER cholesterol content, enhanced HMGCR and SQLE expression and promoting cancer cells death [134, 135]
	Itraconazole	NPC1	Various cancer	Enhanced the immunosuppressive activity of MDSCs to boost tumor growth by suppressing the RIPK3-AKT-mTORC1 pathway and impeding cholesterol synthesis [136, 137]
	Ezetimibe	NPC1L1	Pancreatic adenocarcinoma	Blocked TCR activation of CD8^+^ T cells but also hijacked the intracellular cholesterol from CD8^+^ T cells [138]
	Torcetrapib	CETP	Breast cancer	Downregulate CETP expression [139, 140, 162]
	PF-06446846	PCSK9	CRC	Inhibited PCSK9 expression and promoted the antitumor activity [141]
	Evolocumab	PCSK9	Breast cancer	Blocked the binding of PCSK9 and LDLR [163]
Targeting cholesterol efflux	RGX-104	LXR	Solid tumors	Activated APOE transcription to regress MDSC activity [142, 143]
	LXR623	LXR	High-grade glioma	Reduced intracellular cholesterol via decreasing uptake and increasing export [145, 146]
	SR9243	LXR	ccRCC, CRC	Induced apoptosis [144]
	2-hydroxypropyl-β-cyclodextrin	LXR + HMGCR	Breast cancer	Decreased LXR expression and increased HMGCR expression [147]
Targeting cholesterol esterification	Avasimibe	ACAT1	Prostate cancer	Upregulated E2F-1 signaling pathway to suppress tumor growth and metastasis [148]
		ACAT1	Bladder cancer	Activated PPARγ pathway to impede tumorigenesis [149]
	Bitter melon extract	ACAT1	TNBC	Reduced ACAT1 expression [150]
Combination therapy	Anti-PD-1 + statins	Immunotherapy + HMGCR	NSCLC	Reprogrammed inflamed TME, enhancing therapeutic efficacy of ICB [152]
	ADC + statins	HER2 + HMGCR	Gastric cancer	Modulated of HER2 internalization, thus sensitized inhibition of HER2 [153]
	Androgen, estrogen receptor + statins	Endocrine therapy + HMGCR	Prostate cancer, HR^+^ breast cancer	Inhibited cholesterol production [154, 159]
	AZD5363 + pitavastatin	AKT + HMGCR	TNBC	Impaired SREBP2 activation and dysregulated cholesterol trafficking [122]
	Anti-PD-1, Kras peptide + avasimibe	Immunotherapy + ACAT1	Melanoma, lung cancer	Increased cholesterol level of CD8^+^ T cells and enhanced CD8^+^ T cells effector function, downregulated the number of Tregs [103, 155]
	Paclitaxel, 5-Fu + avasimibe	Chemotherapy + ACAT1	Melanoma, CRC	Increased cholesterol level of CD8^+^ T cells and enhanced the antitumor effects of chemotherapy [156, 157]
	Metformin, valproic acid + statins	AMPK + HMGCR	Metastatic castration-resistant prostate cancer, pancreatic cancer	Regulation of both HMGCR and AMPK, reverted chemotherapy resistance or exerted synergistic anticancer effect [160, 161]

## Conclusion and perspectives

Substantial evidence indicates that cholesterol metabolism plays a pivotal role in tumor development and response to cancer immunotherapy. This review systematically delineates cholesterol homeostasis under physiological conditions and the characteristics of cholesterol metabolic reprogramming in tumors, including enhanced synthesis, increased uptake, and accumulation of derivatives such as and oxysterols. These alterations not only support the rapid proliferation and survival of cancer cells but also profoundly reshape the tumor immune microenvironment.

Cholesterol metabolism crosstalks with key oncogenic signaling pathway, such as SREBP2, LXR, and mTOR, to collectively regulate cholesterol homeostasis. Non-coding RNAs have emerged as critical regulators of cholesterol metabolism, offering new avenues for biomarker discovery and targeted intervention. The accumulation of cholesterol derivatives modulates immune responses, ferroptosis, autophagy, mitochondrial function, cancer stemness, and interferes with DDR and genomic stability. Within the tumor immune microenvironment, cholesterol metabolism regulates the functions of various immune cells, including T cells, macrophages, DCs, and NK cells, exerting multidimensional control that often leads to immunosuppression and promotes immune evasion. These findings underscore the potential of targeting cholesterol metabolism to enhance the efficacy of immunotherapy.

Cholesterol-targeting agents, such as statins, ezetimibe, avasimibe, and SQLE inhibitors, have demonstrated promising antitumor effects in both preclinical and clinical studies. These compounds restore antitumor immunity and overcome therapy resistance by inhibiting cholesterol synthesis, blocking uptake, promoting efflux, or suppressing esterification. Combination strategies incorporating immunotherapy, chemotherapy, or targeted agents have shown improved outcomes in various tumor models. However, the efficacy of cholesterol-targeted therapies varies across cancer types, reflecting tumor-specific metabolic dependencies and microenvironmental influences, thereby highlighting the necessity for personalized treatment approaches.

Furthermore, most current studies focus on individual cell types or pathways, with limited exploration of multicellular interactions and systemic metabolic crosstalk. Future research should prioritize:

1. In-depth investigation into the context-dependent functions of oxysterols and cholesterol-derived metabolites across different cancer types and immune cell subsets.
2. Exploration of the interplay between cholesterol metabolism and gut microbiota, ferroptosis, autophagy, mitochondrial function, and DDR.

Moreover, beyond mechanistic investigations, cholesterol-targeted combination immunotherapy faces several significant clinical challenges. First, pervasive tumor heterogeneity results in highly divergent metabolic profiles across different cancer types and individual patients, thereby limiting the efficacy of universal cholesterol-modulating regimens. Second, long-term administration of lipid-lowering agents may cause metabolic adverse effects, necessitating rigorous safety monitoring and dose optimization in clinical practice. Finally, the optimal dosage, administration sequence, and combination regimens for cholesterol modulators combined with immunotherapeutic agents remain to be validated in large-scale clinical trials. Overcoming these clinical bottlenecks will greatly accelerate the clinical translation of cholesterol-targeted antitumor strategies.

In summary, cholesterol metabolic reprogramming represents a hallmark of cancer with profound implications for tumor immunity and treatment. Targeting cholesterol pathways offers a promising strategy to overcome immunotherapy resistance and enhance clinical outcomes. Future research should focus on translating these mechanistic insights into personalized therapeutic regimens.

## Data Availability

No datasets were generated or analysed during the current study.

## Abbreviations

25-HC: 25-Hydroxycholesterol
27-HC: 27-Hydroxycholesterol
7-DHC: 7-Dehydrocholesterol
7-KC: 7-K etocholesterol
ABCA1: ATP-binding cassette transporter A1
ABCG1: ATP-binding cassette transporter G1
ACAT: Acyl-CoA cholesterol acyltransferase
AFP: Alpha-fetoprotein
AMPK: AMP-activated protein kinase
APC: Antigen-presenting cell
APOE: Apolipoprotein E
CCR7: CC chemokine receptor 7
ccRCC: Clear cell renal cell carcinoma
CE: Cholesteryl esters
CETP: Cholesteryl ester transfer protein
CRAC: Cholesterol recognition/interaction amino acid consensus
CRC: Colorectal cancer
CSC: Cancer stem cell
DC: Dendritic cell
DDR: DNA damage response
EGFR: Epidermal growth factor receptor
ER: Endoplasmic reticulum
FAO: Fatty acid oxidation
FPP: Farnesyl pyrophosphate
GBM: Glioblastoma
GSH: Glutathione
GPX4: Glutathione peroxidase 4
HA: Hyaluronic acid
HCC: Hepatocellular carcinoma
HDL: High-density lipoprotein
HER2: Human epidermal growth factor receptor 2
HIF-1α: Hypoxia-inducible factor-1α
HMG-CoA: 3-Hydroxy-3-methylglutaryl-CoA
HMGCR: HMG-CoA reductase
HP-β-CD: 2-Hydroxypropyl-β-cyclodextrin
HR^+^: Hormone-receptor positive
IFN-γ: Interferon-γ
IPP: Isopentenyl pyrophosphate
LDL: Low-density lipoprotein
LDLR: Low-density lipoprotein receptor
lncRNA: Long non-coding RNA
LRP8: LDLR-related protein 8
LXR: Liver X receptor
MDSC: Myeloid-derived suppressor cell
MHC: Major histocompatibility complex
MIEF2: Mitochondrial elongation factor 2
miRNA: MicroRNA
ncRNA: Non-coding RNA
NK cell: Natural killer cell
NPC1: Niemann-Pick type C1
NPC1L1: Niemann-Pick C1 like 1
OCS: 2, 3-Oxidosqualene cyclase
PCa: Prostate cancer
PCSK9: Proprotein convertase subtilisin/kexin type 9
PD-L1: Programmed cell death ligand 1
PPAR: Proliferator-activated receptor
PTEN: Phosphatase and tensin homolog
ROS: Reactive oxygen species
SCAP: SREBP cleavage-activating protein
SLC25A: Solute carrier family 25 member
SQLE: Squalene monooxygenase
SREBP: Sterol regulatory element-binding protein
TAM: Tumor-associated macrophage
TCR: T cell receptor
TME: Tumor microenvironment
TMEM147: Transmembrane protein 147
TNBC: Triple-negative breast cancer
Treg: Regulatory T cell
